# Adaptation of Clinical Research Staff to Decentralized Clinical Trials and Impacts on the Patient-Centered Experience: Qualitative Interview Study

**DOI:** 10.2196/62947

**Published:** 2025-06-16

**Authors:** Eoin Gamble, Ciara Heavin, Conor Linehan

**Affiliations:** 1 School of Applied Psychology University College Cork Cork Ireland; 2 O’Rahilly Building, College Rd, University College, Cork Cork University Business School University College Cork Cork Ireland; 3 N Mall, Kilbarry Enterprise Centre, Cork School of Applied Psychology, University College Cork Lero the Research Ireland Centre for Software Cork Ireland

**Keywords:** decentralized trials, decentralized clinical trial, DCT, remote trials, clinical research technology, site experience, patient centeredness, patient experience, participant-centric trials, human centered

## Abstract

**Background:**

Industry stakeholders, academic experts, and regulatory bodies emphasize the importance of prioritizing a patient-centered experience in clinical trials to enhance retention, adherence, and trial participation. Concurrently, there has been a notable rise in the adoption of technology-mediated decentralized methodologies for conducting clinical trials. Nonetheless, is a truly patient-centric approach being achieved? The shift to decentralized approaches risks prioritizing operational efficiency and remote data collection over the nuanced and diverse needs of participants. This raises critical questions about whether the current implementation of decentralized clinical trials (DCTs) fully aligns with the principles of patient centeredness.

**Objective:**

Using a qualitative approach, our study aims to understand the perceptions of clinical research staff (CRS) of this transition and its impact on delivering a patient-centered experience.

**Methods:**

A total of 15 CRS with experience of facilitating DCTs were interviewed, and transcripts were analyzed.

**Results:**

Our findings reveal 1 superordinate theme—the quality and frequency of interactions with patients and CRS are limited—and six main themes: (1) increasing demands of the CRS role, (2) creating difficulties for patients, (3) knowing the patient and understanding their experience, (4) impacts on forming and maintaining CRS-patient relationship, (5) difficulty in delivering desired level of support and care, and (6) perceived affects to conduct of a trial. While DCTs offer advantages in improving accessibility, they introduce new complexities that can negatively impact patient engagement, retention, and the CRS-patient relationship.

**Conclusions:**

This study offers insights into the perspectives of CRS on delivering a patient-centered experience in DCTs. It highlights the benefits of DCTs alongside the challenges they present, emphasizing the need for additional support, training, streamlined technology, and resources to help CRS adapt to evolving trial dynamics. To fully realize the patient-centered potential of DCTs, technology and trial designers must recognize the complex interplay of factors shaping the patient experience. This requires collaboration with CRS and patients to address the diverse needs of all stakeholders, prioritizing the broader aspects of the patient experience beyond at-home convenience and data collection.

## Introduction

### Background

Clinical trials play a crucial role in testing new treatments and advancing medical knowledge [1] by evaluating specific interventions on human participants [2], and they are traditionally conducted through in-person visits to health care sites [3]. However, developments in the United States, such as the 21st Century Cures Act, demonstrate increasing legislative support for using technology for facilitating CRS-patient interactions in pharmaceutical research [4,5]. Clinical trials are undergoing a shift, with a growing emphasis on adopting technology-mediated approaches [6,7]. Terms such as “decentralized” [8], “remote” [9], “direct-to-participant,” and “virtual studies” [10] have emerged to describe this approach to trial design and execution. In addition to the fully remote approach, hybrid trials incorporate decentralized and site-based elements [11]. “Decentralized clinical trial” (DCT) has emerged as the unified term to describe this type of trial [12]. For conceptual clarity, this paper uses “DCTs” to refer to these types of trials.

DCTs use digital technologies to facilitate remote patient participation in research as well as data collection and communication [13]. Data collection and assessments are now often carried out in participants’ homes through technology [14], replacing traditional on-site data collection, minimizing physical site attendance, and emphasizing participant-technology interactions over interactions with site staff [15]. This includes integrating technologies such as smart devices, wearables, and sensors for data collection and processing [16]. Electronic patient-reported outcomes are collected using mobile apps or web interfaces [17], while telehealth technology facilitates remote interactions, reducing or eliminating the need for in-person visits [18].

The concept of patient centeredness has become paramount in drug development and clinical trials [19], with patient-focused drug development receiving significant attention from governing bodies [20]. Patient centeredness is defined as addressing the needs of the patient across all phases of a trial, including design, activation, enrollment, data collection, completion, and outcome reporting [21]. This concept emphasizes the patient’s experience during a trial [22] and stresses that the trial design supports the needs and preferences of participants [12]. Concepts such as person centeredness [23] and patient centeredness [24] are directly linked to various components of the delivery of care that contribute to the patient’s overall needs and experience. Key aspects such as different forms of patient well-being [25], satisfaction [26], relationships [27], autonomy [28], support and organizational systems [29], personalization [30], trust [31], and communication and education [32] are interconnected within the centeredness definitions, contributing to a deeper understanding what constitutes a patient-centered experience.

### Objectives

DCTs and patient-centered concepts converge because decentralized methods are increasingly viewed as supportive of patient centeredness [4], contributing to a patient-centered experience [18] by aiming to reduce patient burden and enhance patient experience [33]. DCT technology vendors promote DCT methods and technology as patient first, human centered, built for patients, designed to meet the needs of the patient, and centered around patient needs. Nevertheless, trial participant attrition has reportedly been a common problem in remote studies [34], indicating that there is still work to be done to enhance the patient-centered experience in such trials. As the field progresses, there is an increasing demand for additional research to delve into stakeholders’ perspectives on DCTs [35]. Further investigation has been recommended to explore the effects of decentralized methods on stakeholders’ challenges and patient participation experiences [15,36]. Therefore, it is imperative to evaluate the effectiveness of current practices in achieving this goal. We used semistructured interviews to capture the perspectives of clinical research staff (CRS; principal investigators, study coordinators, and site nurses, ie, individuals who play an integral role in the patient experience during a DCT) on the impact of DCTs regarding the experience of coordinating and carrying out trials and their impressions of how DCTs affect the patient experience. This research investigates challenges in supporting patients, maintaining the CRS-patient relationship, and fostering patient engagement during DCTs. It aims to enhance the understanding of the practical implications of the transition to DCTs from the perspective of CRS and identify how this shift impacts the delivery of a patient-centered experience.

## Methods

### Research Design

We undertook a qualitative study using semistructured interviews to understand the perceptions of CRS transitioning to decentralized technology-mediated methods for conducting trials and the impact of this shift on delivering a patient-centered experience. These interviews enabled CRS to share their perspectives [37] and provide first-hand accounts [38]. Reflexive thematic analysis, as outlined by Braun and Clarke [39], was used to identify and organize meaningful patterns in the data, offering insights into participants’ perspectives and the underlying themes.

### Ethical Considerations

The study was approved by the social research ethics committee of University College Cork (2022-215) on January 20, 2023. Participation was voluntary, and upon recruitment, participants were provided with electronic information sheets and consent forms outlining the study’s purpose and procedures. All participants provided written informed consent before engaging in this research. Personal details were anonymized, and sensitive data were removed from the transcripts. Participants were informed of their right to withdraw from the study at any time. No compensation was provided to the participants.

### Data Collection

The research was conducted between February 1, 2023, and June 30, 2023. Interviews were conducted on the web via Microsoft Teams video call to accommodate participants’ schedules and locations. Each interview was conducted by the first author and lasted approximately 1 hour. The interview guide was developed based on a review of the literature to address the research question (Multimedia Appendix 1). It featured questions drawn from the literature [40] and focused on the patient experience, the staff-patient relationship, and keeping patients engaged during a trial. The research team engaged in discussions to refine and enhance the interview guide. The interviews followed a conversational approach, mixing open-ended [41] and follow-up probe questions [42], with interviewees given the opportunity to elaborate on their experiences through prompts or follow-up questions to explore themes in greater depth, ensuring detailed accounts relevant to the research question. All interviews were recorded and transcribed using Microsoft Teams and checked for accuracy by the first author. After completing 15 interviews, we concluded that we had sufficient data to address our research question.

### Data Analysis

We followed the 6-phase reflexive thematic analysis process outlined by Braun and Clarke [39]. The first author independently coded the data, identifying relevant codes and themes that aligned with the research question. Collaborative discussions were held with coauthors to refine the codes and themes, leading to consensus on the final interpretations. The first phase involved familiarization with the data through transcription, listening to recorded interviews, reading and rereading the transcripts, and noting initial ideas. During phase 2, initial codes were generated by systematically working through the data to identify meaningful patterns related to the research question. Reflection and refinement ensured that the codes were accurate and representative of the data and aligned with the research question. Phase 3 involved organizing the codes into potential broader themes. Further refinement ensured that the themes were clear, specific, and accurately represented the data. Phase 4 entailed reviewing, refining, and evaluating the themes to ensure that they were logically related to each other and aligned with the data. Collaborative discussions supported the identification of overarching themes, subthemes, and 1 superordinate theme that encompassed 3 themes. Phase 5 consisted of continued analysis, culminating in the final refinement of themes, with clear definitions and names assigned to each theme. Phase 6 focused on producing the analysis, with each theme supported by detailed descriptions and quotes, ensuring that the findings were grounded in the participants’ accounts. Examples illustrating key phases of the data analysis process are provided in Multimedia Appendix 2 [39].

### Participants

Table 1 presents an overview of the interviewees recruited for their professional expertise in remote, hybrid, decentralized trial formats and outlines their years of experience conducting clinical trials [43]. The participants’ work included studies sponsored by commercial parties as well as those that were investigator initiated. Participants were recruited from varied sites and roles across different Western countries to ensure that a range of experiences were captured. Each of the trial staff had previously conducted some form of remote trial, directly interacted with patients, and used a range of technologies, including patient apps and diaries, patient monitors, and telehealth. Participants were recruited initially through an established professional network, which proved instrumental in facilitating introductions to clinical research professionals with experience in DCTs. These initial connections facilitated a snowball recruitment approach, where participants referred us to colleagues within their own networks, providing access to professionals with relevant expertise and perspectives on DCTs.

**Table 1:** An overview of the participants interviewed in the study.

Role	ID	Facility	Experience (y)	Location
Senior research nurse	SRN1	Research center	20	Ireland
Principal investigator	PI1	Research center	20	United States
Senior research nurse	SRN2	University hospital research center	5	Ireland
Clinical research coordinator	CRC1	Research center	13	Spain
Clinical research coordinator	CRC2	Research center	12	United States
Clinical research coordinator	CRC3	Research center	4	United States
Clinical research nurse	CRN1	University hospital research center	3	Ireland
Clinical research coordinator	CRC4	Research center	3	United States
Principal investigator	PI2	Research center	25	Belgium
Principal investigator	PI3	Academic medical center	15	United States
Clinical research coordinator	CRC5	Research center	4	United States
Clinical research coordinator	CRC6	Research center	3	United States
Clinical research coordinator	CRC7	Research center	5	Canada
Study coordinator	SC1	Research center	3	Canada
Research assistant	RA1	Research center	3	United States

## Results

**Figure 1:**
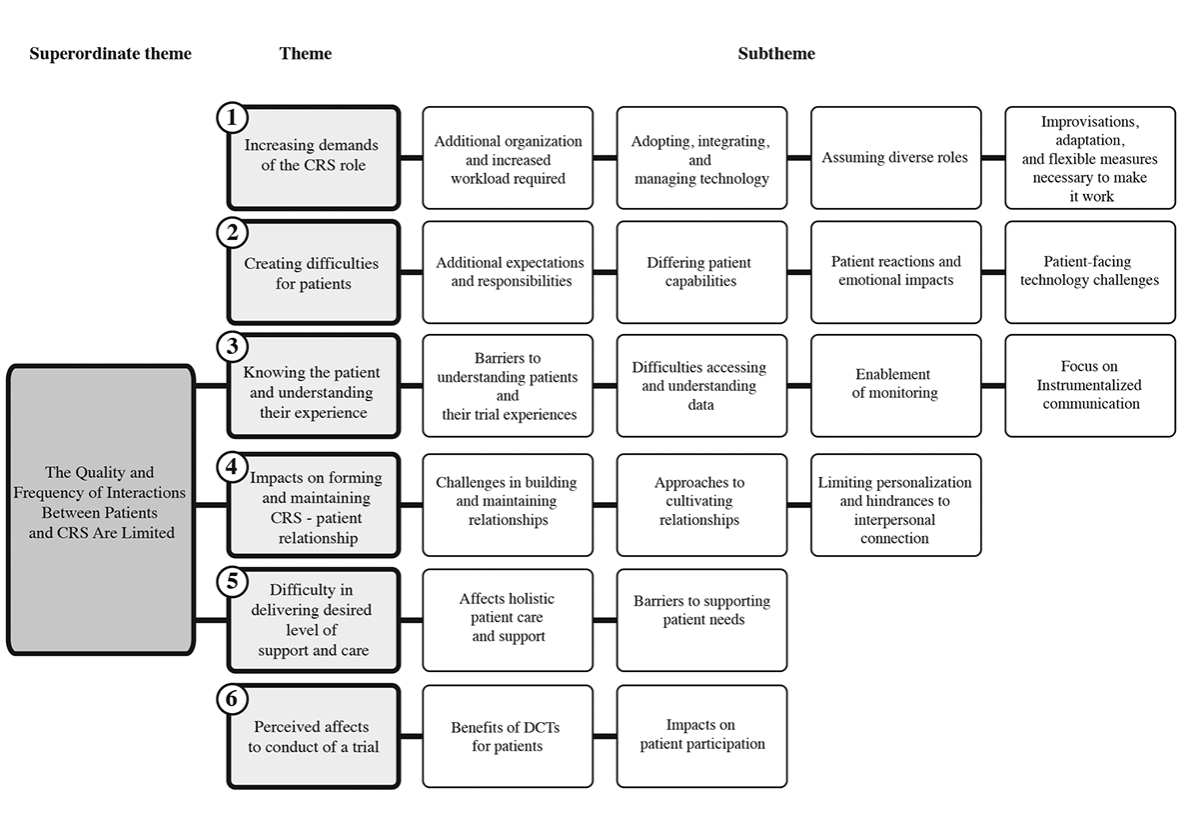
Key themes and subthemes identified in the study. CRS: clinical research staff; DCT: decentralized clinical trial.

Our findings were structured around six themes (themes 3 to 5 are placed within a superordinate theme that identifies how the quality and frequency of interactions between patients and CRS are limited in DCTs): (1) increasing demands of the CRS role, (2) creating difficulties for patients, (3) knowing the patient and understanding their experience, (4) impacts on forming and maintaining CRS-patient relationship, (5) difficulty in delivering desired level of support and care, and (6) perceived affects to conduct of a trial. Each theme has several subthemes, as illustrated in Figure 1.

### Theme 1: Increasing Demands of the CRS Role

#### Overview

This theme reveals the increased demands placed on CRS due to the nature of DCTs. It highlights an increased need for organization and workload management. When discussing their roles, participants highlighted the expanding scope of responsibilities, requiring them to assume diverse roles and implement improvisations, adaptations, and flexible measures to effectively support patients.

#### Additional Organization and Increased Workload Required

CRS noted that remote trials create new tasks and require additional effort compared to face-to-face trials. Impacts include the need to work overtime and deal with late-night calls to address patient-facing technology issues, at times disrupting staff work-life balance:

There have been evenings where my day’s supposed to be 8 to 5 and I have a call at 9-10 o’clock at night with the patient because their diaries aren’t working and it is important for them to do it to start the study drug the next day and they literally cannot, and it is very impactful for us, because we try to have that healthy work-life boundary.

SC1

Participants highlighted the need for heightened organization for successful DCT implementation. Increased efforts are needed during initial visits, involving addressing scheduling issues, aligning site visit resources, coordinating patient contact, and managing appointments. Sustaining communication with remote patients necessitates extensive follow-up and relies on CRS organizing frequent communication via telephone, SMS text message, and email. During a DCT, providing general patient support requires CRS to make a more conscious effort, with challenges particularly evident when supporting older participants who require additional assistance adapting to the technology. Furthermore, there is a need for the trial staff to supplement the technology experience with human interaction to bridge the technology and remote gap:

We try and educate them here as best we can, and sometimes we could be two or three hours going over the same thing.

CRN1

#### Assuming Diverse Roles

A notable shift has occurred in the trial staff’s role, which now includes training patients on technology and providing comprehensive device instructions. The evolved role involves offering technology support as well as follow-up support for seamless technology use. CRS also serve as the primary point of contact for addressing patient issues within the trial setting. Patients directly contact CRS rather than relying on trial support services, and CRS act as an intermediary between patients, sponsors, and trial technology vendors. This expanded responsibility includes addressing technology support, device-related issues, software challenges, and telephone and password problems:

I think clinical trials are changing and adapting to technology and so that’s kind of changed our role in clinical trials and following up with patients and doing phone calls and texting and emails, that’s just part of being a study coordinator now and it can be like very scattered braining for coordinators.

CRC6

#### Adopting, Integrating, and Managing Technology

Participants emphasized the need for additional technical skills to support patients when conducting a DCT. CRS point to an increased workload, exacerbated by the trial technology’s manual and laborious nature; the lack of standardization; and the need to engage with diverse, complex technologies used across the trial portfolios:

So, we have to be very versatile, and we have to know all these systems because these vendors don’t provide us with all that information.

PI2

CRS highlighted the difficulties of misalignment between technology vendors, sponsors, and the practical needs of the trial site. They stressed the importance of vendors being aware of site-specific challenges and integrating site feedback to effectively address technological issues. Furthermore, some CRS felt that technology solutions emphasized administrative processes that overlook the perspectives and needs of both sites and patients, hindering trial efficacy.

#### Improvisations, Adaptation, and Flexible Measures Necessary to Make It Work

CRS discussed the importance of an adaptive and improvised problem-solving approach to ensure the smooth operation of DCTs. Their approach included proactive engagement with patients beyond established trial protocols, such as unscheduled calls, check-ins, tailored email support, and providing handwritten instructions on device use. In addition, CRS described using patient diary entries and data proactively to initiate meaningful conversations in remote interactions. They highlighted flexibility and adaptability as crucial, noting the need to adjust protocol expectations, provide additional telephone support, and schedule remote visits based on patient availability. CRS indicated that they had to rely on instinct and intuition, drawing upon their professional background and experience in their approach to patients. They felt that the limitations of technology meant that they had to develop an awareness of patient patterns and trial-related behaviors:

I’d say it’s mostly my personal approach. Every coordinator works their trials in a different way. I’ve been in research for a few years now, and so I’ve kind of developed that on my own...I think instincts is a good word. It is challenging. You never know. Officially, I use my background and research to kind of gauge when I may need to reach out to patients.

CRC5

CRS indicated that, at times, they needed to use workarounds to overcome the challenges of the provided technologies, remote interactions, and DCT limitations, including patient literacy and connectivity complications. They would circumvent the help desk by directly addressing patient needs, acting as intermediaries, and assuming help desk responsibilities, all to ensure continued patient trial participation:

I tried to be as savvy as I can about all the technology that I can be the help desk. I’m not being paid for it, the help desk are being paid for it, the help desk should be doing it. But I don’t want to put that burden on a patient saying you know, wait and call the help desk. And they don’t have a help desk in the local language, and they have to wait for translator and it’s taking away time from them, which might mean that they will back out. I know that.

PI2

#### Theme 1 Discussion

Remote tools and methods can improve trial efficiency and reduce burdens [44]. However, our findings echo those of Coyle et al [15], highlighting the extra burden on trial staff when using DCT technologies. Our study underscores how the introduction of DCTs has heightened demands on CRS, necessitating additional responsibilities, burdens, and organizational efforts. Our research further underscores how CRS grapple with burdens throughout trials, similar to findings from Rogers et al [35], who identified the challenges that CRS face in providing technical support to patients during DCTs. Our study outlines how CRS roles are evolving in response to DCTs, necessitating the acquisition of additional skills to ensure trial success and effectively support patients. CRS face new challenges and must adapt to the technology focus and remote nature of these trials, at times assuming roles for which they may not be formally qualified. Rogers et al [35], who reviewed how CRS face challenges in learning to manage trials using new technologies [4], suggest that trial site teams may not have the skills to support digital clinical trials. Our findings suggest that CRS are adjusting to the demands of DCTs, a process that requires additional time and effort to ensure trial success. CRS demonstrate adaptability and resourcefulness in response to inadequate existing structures, using workarounds and instinctive strategies. Our findings indicate that DCTs do not fully cater to CRS needs during the trial and may restrict their ability to perform their roles as they envision them. However, these necessary adaptations may bring about negative consequences and disrupt workflows, have unintended side effects, change trial approaches, and lead to potential adverse impacts.

### Theme 2: Creating Difficulties for Patients

Theme 2 focuses on CRS perceptions of the challenges encountered by patients participating in DCTs. This theme discusses the additional expectations placed on DCT participants, their varying capabilities, the emotional impact of being part of a DCT, technology challenges, and the multifaceted nature of challenges within the decentralized setting.

#### Additional Expectations and Responsibilities

CRS discussed the growing expectation that patients will assume more responsibilities in DCTs compared to traditional trials. Participants discussed how patients are expected to undertake tasks such as frequent diary entries, which can become overwhelming. Patients are expected to manage trial-related technology tools, with technology vendors assuming that patients have a comprehensive understanding of the technical hardware and software:

To just give them a little bit of information and give them a little bit of training like, hey, this is on you, now we’ll just check on you every now and then. That’s very much like we just push you out there to the wolves and we’re going to just let you sink or swim. Even though that’s not the case. But that’s how they may feel and that’s how it feels to us as well.

CRC3

#### Differing Patient Capabilities

CRS noted challenges in supporting patients from specific demographic groups, particularly regarding their varying comfort levels with technology. They observed a lack of familiarity and technological literacy challenges, especially among older participants, which posed barriers during a DCT. The disparity between technology solutions and patients’ technical proficiency impacted their ability to operate devices and participate effectively in DCTs:

Some of our patients have a lot of difficulties using technology. It’s insane. The challenges that they face. And I have been on the phone with one person for around 45 minutes trying to walk that person through how to get back into that application on their study device because they had no idea what they were looking at...it’s challenging for them, and they don’t understand how to use it.

RA1

#### Patient Reactions and Emotional Impacts

Participants described the emotional impacts on patients due to the decentralized and technology-mediated nature of DCTs. Frustration with these trials influences the overall patient trial experience and can even lead to withdrawal of consent. CRS observed that patients, particularly older adults who face heightened demands due to the nature of DCTs, experience emotional strain, leading to feelings of being overwhelmed, diminished confidence, anxiety, and increased stress:

We have had patients, if they become frustrated with their piece of technology, specifically the example that comes to mind is an electronic diary like patient-reported outcomes. If they do become frustrated with that, for whatever reason, if they have technical issues that they have to contact support a lot or have to contact the site a lot, they withdraw consent.

CRC2

CRS noted that the nature of DCTs could undermine the development of trust with patients. The limitations of, and the absence of interpersonal interactions in, technology-mediated communication can hinder the trust-building process between CRS and patients, contrasting with on-site trial experiences. Consequently, patients may feel more like trial subjects than individuals receiving personalized care. CRS highlighted how some patients had indicated that they preferred conventional trial methods, which exacerbates the challenges faced by trial sites:

You wouldn’t have the same level of trust as, say, patients that are coming on site, because obviously they don’t build or develop a relationship with the research nurse and the same with the doctor.

SRN1

Conversely, some CRS highlighted how DCT experiences with more comprehensive remote monitoring technologies can reassure patients, positively impacting their well-being during the trial. Patient perception and awareness of efficient trial staff monitoring provide reassurance. Regular informed data reviews with CRS can also reassure patients, boosting their confidence and trust in the CRS and technology used as part of a DCT:

But with the patients with the digital devices, they know they’re being watched every day. They know they’re being monitored every day. So, they have this assurance that they feel confident that they’re okay. There’s somebody watching them, someone looking out for them...I think primarily, the patient feels looked after and has great reassurance of being monitored.

SRN2

#### Patient-Facing Technology Challenges

CRS discussed the challenges that patients face with trial technology, which make the DCT process harder. These include difficulties with device use, connectivity problems, malfunctions, usability concerns, and Wi-Fi–related issues, leading to increased burdens and hindrances for patients and CRS during the trial experience:

I have a patient that we did so much calling back and forth and emailing back and forth over weeks with IT before the resolution was that she just had to drive into town every day. Twice a day to do her diaries to be compliant.

SC1

It’s the layout on the screen and the buttons themselves that have been a slight problem as well...it doesn’t always transform from a desktop to a mobile screen properly, which also is a huge confusion for patients.

CRC7

#### Theme 2 Discussion

Advocates for DCT tools and techniques emphasize their potential to alleviate patient burden in trials [44]; for example, Dorsey et al [45] suggest that data collection becomes passive for patients using DCT technologies. These technologies can also allow CRS to monitor patients’ symptoms and provide informed feedback, which can reassure patients. However, similar to Cummins et al [46], our study suggests that DCTs impose additional expectations and responsibilities on participants. Previously, Moser et al [47] suggested that personal, emotional, and psychosocial factors significantly influence patient attrition in clinical trials. Trust is a pivotal component of both person-centered care [48] and patient-centered care [31]. It facilitates patient participation in research [49] and enhances retention [15,50,51]. Our findings highlight several adverse emotional impacts observed in patients during DCTs, including feeling overwhelmed and frustrated, with a notable impact on patient trust. Our findings challenge the suggestions that DCTs reduce patient burden and promote a patient-centered experience and indicate the potential for negatively impacting patient retention.

### Superordinate Theme: The Quality and Frequency of Interactions Between Patients and CRS Are Limited

#### Overview

We identified an overarching superordinate theme (Figure 1) based on consistent observations made by CRS. The limited quality and frequency of interactions between patients and CRS, stemming from the remote, technology-mediated nature of DCTs, emerged as a central organizing concept in our analysis. This limitation impacts several important components of patient-CRS interactions, serving as a primary obstacle to effective patient management, support, understanding, and care. Three main themes were identified within this superordinate theme, and these are outlined in the following subsections.

#### Theme 3: Knowing the Patient and Understanding Their Experience

Theme 3 discusses how carrying out a trial in a remote or decentralized manner has implications for how CRS understand patients and their experiences. Our data highlight the efforts needed to overcome barriers to comprehending patient experiences, accessing relevant data, enabling effective monitoring, and the instrumentalizing of communication.

#### Barriers to Understanding Patients and Their Trial Experiences

CRS noted that the lack of direct patient oversight in DCTs limits their ability to monitor and understand patients and their experiences throughout the trial. The constraints imposed by DCTs contribute to challenges in understanding the patient’s experiences beyond protocol adherence as well as hinder timely assistance and support. Unlike on-site interactions, DCTs provide fewer insights into patient experiences, and CRS face challenges in monitoring medication adherence, which they perceive may be carried out more effectively during traditional, in-person visits:

It’s very difficult, to be honest, you don’t have the same visibility of how compliant a patient is either. It’s not only around compliance with the study, if they were taking medication or that kind of thing, it’s easier for them to come into clinic to have oversight of that as well.

SRN1

Remote interactions in DCTs present challenges in obtaining important patient information and understanding. Prompts from the patient are needed to understand when they are having issues. The absence of informal dialogues and in-person interactions hinders CRS awareness of pertinent patient details, affecting the evaluation of patient conditions and the timely recognition of changes in patient conditions:

You’re able to pay attention to the little things in a patient when they come into the office. They may seem a little bit more tired than the last time you saw, or you may notice that their skin may look different, or their veins when you normally you blood draw that their veins don’t look the same as they did, you know. So you pay attention to the very little things that can possibly save a patient’s life.

CRC3

CRS depend on patient questionnaires to identify issues and comprehend patient needs. However, the types of data collected compromise CRS awareness of patient conditions and hinder informed discussions, evaluations, and individualized feedback, posing challenges in tailoring support. The limitations in technology-driven reports diverge from traditional trial site insights, impacting the depth of support provided. The remote and technological nature of DCTs delays CRS recognition of patient needs and hinders the delivery of comprehensive support. CRS also encounter challenges determining when to initiate patient contact and providing timely support beyond trial protocol expectations and data collection requirements. Establishing communication with remote patients is challenging and complicates the trial staff’s role, leading to additional stress. It requires concerted efforts to connect and coordinate schedules between CRS and patients, and it is common for patients to be unresponsive to calls or messages.

#### Difficulties Accessing and Understanding Data

The participants highlighted the complexity involved in analyzing, identifying, and understanding the collected trial data, which made related tasks laborious. Limitations in technologies for data observation and analysis in DCTs can lead to delayed issue detection, burdensome data processes, and insufficient reports or insights to support patients. Some CRS reported relying on informal methods, such as using Microsoft Excel, to analyze patient data and identify changes or symptom patterns. They also noted a time lag in patient compliance awareness and challenges in acquiring real-time data:

Every vendor portal is very different, for some of them you literally have to click into every single diary to see that data and then go back and refilter. And it’s a very cumbersome process. Even to get a week’s worth of data, especially when you’re looking at multiple and different results from patients, there’s no easy way to export that data.

SC1

#### Enablement of Monitoring

CRS praised the advancements in DCT technology for enabling remote patient monitoring, including tracking patient progress, medication adherence, and symptoms. This capability empowers CRS to identify concerns and manage patients. In addition, technology centralizes data and documentation, streamlining access and facilitating longitudinal comparisons of patient information. Furthermore, some technologies enhance staff-patient interaction by providing insights into patient patterns, thereby informing and enabling more effective communication. Notifications enable timely interventions, and data readings enable analysis, both ensuring patient safety and well-being through remote monitoring:

The person you’re ringing can see your blood pressure, can see your oxygen level, can see your weight, can see pulmonary artery pressure. So when they tell you, No. You know your blood pressure is okay. Your weight is okay. You’re basing it on fact, and they know you can see it, so that’s it. They’ve confident in what you say, and they’re reassured by what you say.

SRN2

#### Focus on Instrumentalized Communication

CRS highlighted that patient contact in a DCT is primarily prompted by formal problems, such as compliance concerns, irregular diary entries, and compliance or data discrepancies, which then necessitate site follow-up. Our data highlighted how remote visits focus solely on a business-only and data collection approach. Structured assessments during remote visits, centered around specific questions, reinforce this formal, data-centric dynamic, resulting in a lack of casual conversation and personal connection opportunities and restricting the interpersonal relationship between CRS and patients:

You know, you talk about business, and you get through what you need to get through and then the visit is usually terminated...Usually the visit concludes at that point as opposed to an ongoing banter back and forth about whatever.

PI1

But when you’re on the phone, you have exact questions that you’re going to ask them. There really isn’t any time for chitchat.

CRC6

#### Theme 3 Discussion

Research has shown that effective patient care depends on the trial staffs’ ability to understand and respond to individual experiences [52]. Fogel [53] outlined the significance of the trial staff’s ability to maintain awareness of participants’ feelings throughout the trial process. Our research suggests that DCTs pose barriers for CRS in understanding patients and their trial experiences, stemming from the absence of direct patient oversight and the constraints of technology-based interactions. These limitations hamper the trial staff’s ability to fully understand the patient’s needs. The crucial role of CRS in supporting patients’ experiences and the gap in understanding patients’ experiences in DCTs leads to a disparity in effectively supporting patients throughout such trials. CRS discussed how DCTs necessitate a business-oriented, data collection approach during remote patient interactions, particularly during communication driven by trial protocols. They noted how a lack of patient compliance is the primary trigger for reaching out to patients outside of established protocols. These instrumentalized communication approaches may diminish their sense of autonomy, and this depersonalization can adversely affect patient satisfaction, motivation, trust, patient outcomes, and participation. It is important to note that our study emphasized how some DCT technologies can facilitate enhanced patient monitoring. These findings resonate with those of Coravos et al [54], showcasing how DCT technologies enable real-time symptom alerts [55,56] and provide pertinent patient data and information on study activities.

#### Theme 4: Impacts on Forming and Maintaining CRS-Patient Relationship

Theme 4 discusses the impacts of DCTs on forming and maintaining CRS-patient relationships. It examines the dynamics surrounding these relationships during DCTs, addressing challenges in connection building, developing interpersonal bonds, and the various ways in which these relationships are cultivated.

#### Challenges in Building and Maintaining Relationships

Several challenges were identified in building and maintaining relationships, including limitations in developing connections with patients due to the remote nature of DCTs. CRS highlighted that the absence of face-to-face interactions at trial sites poses hurdles in establishing meaningful connections. Unlike traditional on-site trials, DCTs present more significant challenges due to limited interactions, removing opportunities for creating positive rapport and impeding the formation of the typical bonds developed during on-site trials. Participants also discussed issues with DCT technology functionality, pointing to a lack of dedicated mechanisms to support the interpersonal dynamic between the trial staff and the patient:

My biggest challenge is just being able to form that relationship and letting the patient know, making them feel comfortable enough to talk to us as often as they would need to and to be open with us.

CRC5

#### Approaches to Cultivating Relationships

CRS noted that jointly navigating technology challenges with patients fostered camaraderie through increased interaction during remote technological issues. Increased interaction with patients during their health challenges allows CRS to provide assistance, strengthening the CRS-patient relationship. Furthermore, trial data and readings collected using patient-facing technology provide CRS with patient insights and serve as a focal component, contributing to personalized insights and calls and, in turn, supporting relationship development. Relationships are further enhanced by personalizing patient information, proactively providing tailored support notes, engaging in conversations about personal matters, staggering interactions, and showing genuine interest:

You’d have a bond with all the patients, but it would be stronger with the patients that would be in bigger trouble because the readings are gone off. But because you sort out that, and you either sort out their medication or get them into hospital. They trust you and therefore the bond it is stronger.

SRN2

#### Limiting Personalization and Hindrances to Interpersonal Connection

CRS discussed how patient interactions and technology limited personalization and hindered interpersonal connection, resulting in an impersonal and generic patient experience. They discussed various issues affecting the connection and bond with their patients, which could potentially lead to patient attrition. They highlighted the limited opportunities to build rapport, develop lasting connections, and enhance interactions, particularly due to the challenges of maintaining a connection remotely without past interactions or shared experiences with patients. By removing in-person engagement, DCTs and technology-driven experiences may leave patients feeling a lack of human care, fostering a perception of trials as cold and impersonal. Simultaneously, SMS text message and telephone interactions, prioritized for efficiency, may further diminish emotional understanding due to their brevity:

Ideally, we want to provide the best medical care possible, whether we’re around or not, so we want patients to have a good experience with the technology. We want them to have a good experience with the medication that we’re providing to them and with whatever telehealth visits that we are providing. But it is challenging because we don’t have a chance to form that sort of bond that we would with our traditional patients that we see more often.

CRC5

CRS noted that DCTs can contribute to a sense of estrangement for both CRS and patients. Remote patients risk being reduced to numbers in the system, and CRS may overlook them due to on-site priorities, potentially leaving them feeling neglected. CRS also expressed concern about the absence of individualized attention for patients and the challenge of providing personalized care in remote trials:

Sometimes I’ve even caught myself doing it where I’m like, who even is this person that I have to contact today? Like, I don’t even recognize their name because I could tell you about that person when they came into the site four months ago. But now they kind of just fade into a number in the system...I don’t want to say dehumanizes, but it definitely takes away from that relationship when they go remote.

RA1

CRS discussed how questions arise regarding patient concerns for their data, utility, and overall contribution to the trial. Patients’ perception of their data disappearing into a void and their limited understanding of where the data they provide go and how these data are used raises questions about the value and meaning of their participation, as does the limited positive feedback to patients through technology. CRS acknowledged the importance of on-site face-to-face interactions, which allow for direct communication, enabling them to assess patients’ comfort levels, provide tailored support, build connections, and focus on their needs, fostering a humanized approach to care. Some technologies support CRS, and for those with access, video platforms help replicate the in-person experience, enabling the delivery of person-centered care remotely.

#### Theme 4 Discussion

The relationship between CRS and participants is crucial for fostering positive trial experiences [57]. Similarly, the relationship between patients and their health care professionals is fundamental to the concept of patient-centered or person-centered care [27]. Relationships are not merely psychological concepts; they are integral to human motivation and self-determined behavior [58] and serve as a cornerstone for retaining participants and sustaining their motivation throughout a trial [59]. Mohr et al [60] highlight the correlation between CRS fostering personalized relationships with patients and increased treatment adherence. Personalization can enhance relatedness [58]. Fogel [53] highlights the significance of personalizing clinical trial interactions to support a successful trial’s execution. Similarly, de Silva [61] underscores the importance of personalization as a critical component of the person-centered care concept. Our findings highlight how DCTs can negatively impact the formation of relationships and interpersonal connection between CRS and patients and restrict opportunities for personalization and connection. DCTs face considerable hurdles in both motivating [59] and maintaining a patient-centered experience due to the indispensable role of human connection in care [62] and staff traditionally enabling the patient experience [40]. The challenges in relationships, interpersonal connections, and personalization stem from technological limitations, remote constraints, and limited human connection. DCTs struggle to replicate the vital human element necessary for delivering patient-centered trial experiences. As a result, DCTs will face challenges in adherence as well as in effectively motivating, engaging, and retaining patients.

#### Theme 5: Difficulty in Delivering Desired Level of Support and Care

Theme 5 explores the challenges for CRS in delivering the desired support and care to their patients. This theme underscores the difficulties that CRS face in achieving their intended level of support, highlighting intricate and multifaceted challenges in addressing diverse and individualized patient needs.

#### Affects Holistic Patient Care and Support

CRS voiced concerns about the patient centricity of DCTs, noting a misalignment with patient-centered principles. Some believed that these trials prioritize data and project management over patient needs, hindering a patient-centered experience. The limitations of DCTs restrict the trial site’s ability to support a patient-centered experience compared to traditional on-site approaches:

They don’t get the patient-centric experience. They don’t. That’s what I’m trying to tell you. They don’t. They’re just a collecting tool for the industry that we are facilitating, and we have to explain that this is all very cool. Distant...Again, as I started this conversation, these things are set up from a data management and a project management point of view, never from a site or a patient’s point of view...So we are calling it patient centric. But the device is literally shoved down my throat, and then they go, “Is it patient centric?”

PI2

CRS noted challenges in maintaining patient care in the DCT setting, which impacted clinical support and posed potential barriers to fulfilling care roles. Some CRS did not view DCTs and remote approaches as synonymous with patient care. Delivering care through short, remote calls using technology was considered challenging. CRS expressed uneasiness about perceived care levels, with concerns that patients might question the care provided in a DCT. On-site interactions were deemed more impactful for personalized, in-person engagements, contrasting with remote DCTs, which raised concerns about patient safety and the appropriate use of investigational products, requiring a delicate balance in maintaining patient interactions:

With the traditional setup, it’s obviously a higher level of patient care because you’re physically bringing the patient in.

CRC2

DCTs and remote settings hinder CRS from fully understanding and addressing patients’ emotional and psychological well-being. Participants revealed challenges in offering emotional support to patients and perceived that patients may interpret this as a lack of empathy, validation, and acknowledgment from the site, potentially straining empathetic connections:

It can come off as we don’t care about the patient emotionally, the sponsor doesn’t really care about the patient, doesn’t care about them as an individual, they are just seeing them as our participant. And obviously that’s not true. But when you don’t put in those measures to effectively monitor someone’s emotional state or even just ask them how they’re doing. It can come off as extremely cold and just unfeeling.

RA1

#### Barriers to Supporting Patient Needs

CRS face various challenges in providing technical and troubleshooting support. Among these are inadequate site support capabilities for executing remote technical assistance, which make it difficult for CRS to guide patients remotely and lead to delays in resolving support issues:

So definitely trying to help someone remotely with technology is one of the most frustrating things I think I’ve ever faced because a lot of the times I can’t see what that person is looking at. I log into my account on the sponsor’s website, my view is completely different from a patient’s view. So the patient might be looking at a home screen that looks completely different than mine and so I have to figure out, how do I get them to what I need them to get through when I can’t even see what the heck they’re seeing, whereas being physically on site I can see everything.

RA1

Inadequate help desk support presents challenges for patients, who experience poor response times to their issues, leading to frustration for both patients and CRS. As a result, CRS are often forced to take on help desk roles or refrain from using the help desk altogether. This results in a misalignment between the support provided and patient requirements. Some CRS also expressed frustration at being expected to perform technical support tasks for which they are not qualified. These inherent challenges contribute to trial staff frustration amid the myriad of issues they encounter:

Being an IT worker that I’m not qualified to be, trying to walk them through their technology, walk them through repairing things.

RA1

Our study highlights the challenges of a reactive trial staff support model in DCTs, in which patients typically initiate contact during challenging personal and technical situations. Some CRS described this model as reactive in nature, resulting in crisis management rather than proactive intervention. CRS identified challenges in comprehending and addressing patient support needs, often experiencing delays in recognizing the intricacies of patient needs. Participants discussed challenges with timely communication and follow-up support mechanisms, compounded by limited patient availability and unresponsiveness. These factors delay patient communication and hinder the trial site’s ability to provide timely support:

It’s definitely harder to recognize when a patient needs support when we’re doing remote interactions.

CRC6

Furthermore, CRS encounter challenges during onboarding and initially educating patients regarding trial requirements, particularly in ensuring their competence with technology, processes, and tools. Participants noted that the onboarding process can be challenging and time consuming, remote education further complicates support, and real-time assistance is lacking. Overall, CRS emphasized the effectiveness of on-site support, highlighting its advantages in providing comprehensive and interactive assistance to patients compared to remote methods.

#### Theme 5 Discussion

At the core of the patient-centered trial concept is the need to address each patient’s individual needs [12]. Research by Moser et al [47] highlights the importance of addressing patients’ personal, emotional, and psychosocial aspects to enhance retention in clinical trials. Similarly, Ferrell et al [63] emphasize the critical role of compassion and nonphysical support for trial patients. Furthermore, Keshtkar et al [64] demonstrate that empathy enhances patient satisfaction with their care and contributes to favorable patient outcomes. Interconnected support components are crucial in DCTs to ensure successful troubleshooting, uphold data integrity [65], and maintain clinical support aligned with good clinical practice [66]. In addition, fostering patient competence is essential for retention [59] and continued motivation [58]. Johnson and Marsh [67] highlight the challenges involved in supporting the practical trial needs of patients, and Polhemus et al [68] support patients’ technology needs. Our study supports these insights and outlines the challenges that CRS face in supporting patient needs within a DCT context, emphasizing the limitations imposed by site capabilities and technology in offering adequate support in their absence.

### Theme 6: Perceived Affects to Conduct of a Trial

Theme 6 uncovers the impacts of DCTs on trial conduct, exploring the dual nature of their benefits and challenges. It examines how DCTs influence crucial aspects such as patient retention, engagement, responsiveness, and compliance and highlights benefits such as ease of participation, convenience, flexibility, and patient empowerment.

#### Benefits of DCTs for Patients

Some CRS outlined several ways in which DCTs benefit patients, enhancing their overall experience. These included ease of participation and increased eligibility due to reduced barriers. By minimizing logistical burdens, such as travel to clinic visits, DCTs help patients alleviate stress and avoid exposure to potential hospital-acquired infections. CRS perceived that patients appreciated being in the familiar environment of their homes, which is especially beneficial for those considered vulnerable, with family members and caregivers also experiencing reduced travel demands. In addition, some CRS viewed DCTs as enabling greater participation and convenience, especially among younger patients who welcome the reduced need for on-site visits and tend to prefer digital diaries over paper diaries:

It improves eligibility because it eliminates a lot of the logistical barriers.

PI3

Moreover, some CRS perceived DCT technologies as empowering for patients, involving them directly in health monitoring and fostering control and understanding of their health through measurements and insights, instilling a sense of ownership in patients. Remote health monitoring enabled by certain DCT technologies allows CRS to collect factual and relevant patient data, which are then used to enhance the support they provide patients. Regular data reviews reassure patients and improve monitoring and support effectiveness. Some CRS felt that DCTs were seen to enable flexible and efficient trials, saving time through quick assessments, remote communication, and information sharing, while telehealth was viewed as effective for assessing patient concerns and reducing paper-based processes, thereby improving trial efficiency:

They have control, they have power, and where maybe traditionally you feel you have no power, that you go to doctor, he gives you tablets, but there might be no explanation. You don’t know the mechanism of what’s going on, where we go to pains to say why we’re increasing tablets, why we’re decreasing tablets, and you’re able to show them the next time they’re in, or if they’re in hospital the next day, how the drug reacted and how their pressures came down. So they can physically see how things are working.

SRN2

#### Impacts on Patient Participation

CRS outlined the multifaceted impacts and challenges affecting patient trial participation. These include reduced patient comfort in opening up during conversations and a reluctance to share important health updates or concerns. Compliance with the trial protocol is impacted, with missed diary entries, for example, necessitating significant site efforts to re-engage patients. CRS also noted compliance being affected by technology issues and slow site responsiveness. Furthermore, delays in accessing patient data affect CRS ability to identify noncompliance. Conversely, on-site care and monitoring can aid patient adherence to protocols. CRS perceived lower patient engagement, responsiveness, and commitment levels in DCT settings. The DCT approach may impact patients’ understanding of study details and their willingness to participate in future trials because some patients decline trial involvement due to technological reluctance and their unfamiliarity with site staff. DCTs also impact accountability, responsibility, and patients’ understanding of their roles, with obstacles such as forgetfulness and non-responsiveness further impacting participation. In addition, ongoing consent and retention may be affected, and challenges faced during DCTs could lead to higher dropout rates:

I know that if I didn’t put that effort in, which isn’t expected of us, that we would have noncompliance or patients dropping out or lost to follow-up because it’s hard to work with.

SC1

#### Theme 6 Discussion

Minimizing patient burden is crucial for retaining patients in trials [50], and DCTs promise to reduce patient burden [7]. Logistical hurdles such as travel time and distance to trial sites pose significant barriers to patient participation [69]. DCTs address these challenges by offering convenience and flexibility [65,70]. Our research suggests that CRS perceive DCTs as providing enhanced ease of participation, reduced logistical burdens, time savings, and, in some cases, increased patient control over their health—findings that align with previous research. The benefits inherent in these measures give patients autonomy, allowing them flexibility, convenience, and empowerment—enabling them to exercise volition over their health and ownership of their trial experience. The importance of supporting autonomy cannot be understated because patient autonomy is regarded as an ethical mandate in medical research [71] and a vital factor in sustaining motivation [58]. However, our study also suggests that CRS perceive DCTs as having detrimental effects on the conduct of trials, affecting retention [72], accountability [71], and understanding of patients [73], all of which are crucial to successful clinical trial outcomes.

## Discussion

### Overview

We conducted 15 expert interviews and performed reflexive thematic analysis to explore CRS perspectives on executing DCTs. By focusing on CRS experiences and perceptions, we developed rich insights about the types and nature of additional tasks needed to make this type of trial work and how remote participation affects the CRS-patient dynamic and patient experiences. The findings highlight the heightened demands placed on CRS within DCTs. We offer several insights into the implications of transitioning to DCTs and their impact on clinical trial practices.

### Implications for Trial CRS

Our data underscores the evolving working dynamics and conditions, increased demands, and an expansion of the CRS's role to include technology support. CRS must adjust to new ways of supporting, monitoring, and interacting with patients remotely. These changes necessitate additional expectations and require increased time and effort from CRS, mirroring challenges observed in other contexts [74] when remote, technology-mediated approaches replace face-to-face interactions. The evolving technical expectations of CRS within DCTs may require a reimagining of their roles and necessitate formal adjustments to role expectations. Consequently, it is imperative to reassess and update training and professional development programs to ensure that CRS are equipped with the necessary skills for navigating the technical challenges of DCTs [4]. This re-evaluation underscores the importance of enhanced training in remote communication and support, IT, and digital proficiency. These programs can support successful practice in DCTs and prepare CRS for future DCT advancements. Our observations indicate that, despite retaining responsibility for patient care and trial delivery, CRS experience a loss of control and diminished ability to influence patient behavior compared to traditional trials. This loss of control and influence hampers their ability to support patients and fulfil their roles to their desired standards. Our data show that CRS adapt to the challenges of conducting a DCT and find ways to make it work. This adaptability is crucial for maintaining patient engagement, upholding care standards, ensuring operational efficiency, and achieving trial success. However, as observed in other contexts [75], the integration of new technology-mediated approaches into established practices can have hidden or unintended consequences for CRS. These may include increased cognitive load, potential for errors, reduced job satisfaction, and burnout.

### Implications for DCT Design

A concern highlighted with conducting clinical trials in a decentralized manner is the potential for negative impacts on critical factors essential to successful trial execution. CRS identified that patient engagement, responsiveness, commitment, retention [72], accountability [71], and compliance [76] may all be adversely affected within DCT contexts. If this is indeed the case, it necessitates strategic reconsiderations from a trial design and technology choice perspective. Our observations suggest that while DCTs offer the potential to overcome some challenges associated with traditional on-site clinical trials, such as improving accessibility and convenience [35,77] for patients, they introduce new complexities that CRS and patients must overcome, including challenges with support, communication, understanding patient experiences, and engagement, as well as technical complexities and the burdens and expectations placed on patients. Our research suggests that transitioning from traditional on-site trials to DCTs requires rethinking the design of patient engagement practices. Approaches that are practical in traditional, in-person trials may not be suitable for DCTs. The CRS-patient relationship plays a pivotal role in patient-centered and person-centered paradigms [27,57] and is an important component in successful trials [59]. Furthermore, CRS perceived that additional aspects such as personalization [30], trust [31], well-being [25], and satisfaction [26] are also impacted. These elements are integral to a positive patient experience and play a significant role in achieving favorable outcomes. DCTs have the potential to significantly improve the access, inclusivity, and diversity of trial participants [78]. Our observations reveal that DCTs offer an instrumentalized, mechanistic, and transactional experience for patients, which, based on the evidence, affects personal interactions and fosters a detachment from CRS. CRS discussed various challenges faced by patients, particularly older adults. These challenges may hinder the participation of certain groups and, in turn, exacerbate disparities in trial representation.When envisioning the future of DCTs and trial design, emphasis should be placed on prioritizing greater patient choice and flexibility to accommodate the crucial need for patient autonomy [58,71]. We speculate that a more flexible and adaptive trial could be instrumental in addressing patient challenges related to competence issues, technological capabilities, accessibility factors, and the necessity for human connection, while also providing patients with choices to meet their individual needs.

### Implications for Technology Design

Our observations indicate that there should be a careful consideration of both trial site and patient-facing technologies to better understand how they can address the challenges outlined in this paper. Our data suggest that current DCT technologies, which primarily focus on data collection, would benefit from focusing more on supporting the experience of patients. Technology that supports an understanding of patients’ experiences and creates conditions for personalization [30], trust [31], well-being [25], and CRS-patient relationships [27,57] would enhance the overall experience. Resolving patient issues, such as ease of use, accessibility, and reliability, can reduce barriers to acceptance and enhance adoption and use [79]. We suggest applying a theoretical and behavioral science–grounded approach to inform strategic directions for patient-facing technology, guide interventions, shape design, and define solutions tailored to meet patient psychological needs and supporting behaviors [57,80]. Our results highlight the critical need to involve diverse stakeholders in future design processes to develop more comprehensive and inclusive technologies, with benefits in doing so [81]. Stakeholders, including technology vendors, designers, behavioral scientists, regulatory bodies, patients, CRS, and pharmaceutical companies, must continue engaging with the complexity of the challenge. Leveraging our findings, designers can focus their efforts on addressing the issues outlined, ensuring that technological solutions align with both trial staff and patient needs and support optimal experiences and effective technological solutions for all concerned.

### Limitations and Future Research

The shared themes and insights from our interviews are not intended to represent universal applicability. Rather, they provide a foundation for understanding and serve as a springboard for further research and discussion. This research focuses exclusively on the perspectives of trial staff involved in DCTs. While the analysis of CRS experiences and observations is valuable for providing insights into trial conduct, impacts, and perceived patient experiences, future research should incorporate the perspectives of patients, pharmaceutical companies, and technology vendors to provide a comprehensive view of DCTs. Technology is a key enabler of DCTs. However, these trials also depend on nontechnological elements, such as home health visits and other patient-centric approaches, that support the model but were not addressed in this paper. Future research may offer promising opportunities for both academia and practice. We recommend expanding on stakeholder perspectives, particularly through a further investigation of how DCTs are experienced and perceived by patients. In addition, focusing on specific technological experiences may offer a better understanding of the perspectives surrounding these technologies. Moreover, future studies should explore integrating behavioral science into the DCT design approach to better support participants’ experiences and practitioners’ efforts to deliver more patient-centered trial experiences. Research should also investigate strategies to support CRS in DCTs, including tailored training programs, help desk assistance, and support structures that address the unique challenges of remote trial environments. Finally, investigating lessons from successful technology adoption in health care could provide valuable insights into overcoming barriers and leveraging enablers for technology integration in DCTs, helping the clinical trial ecosystem adopt proven strategies for success.

### Conclusions

First, we must acknowledge that one of the primary goals of clinical trials is to collect patient data while navigating complex challenges such as regulatory compliance, patient burden, study objectives, safety, scalability, protocols, and ethical considerations. Despite the many benefits of a remote approach, such as improved accessibility and data collection, DCTs have fundamentally altered trial dynamics for both patients and CRS. Approaches that work in traditional face-to-face trials may be less effective in the context of DCTs, leading to unique challenges. CRS play a crucial role as indispensable mediators, harmonizing the relationships between patients, support systems, and trial protocols. As workplaces evolve with increasing technological mediation, CRS face challenges that inevitably impact their working dynamics and, potentially, their performance. To adapt effectively to DCTs and the evolving trial dynamics, CRS need adequate support and resources, such as enhanced training, effective change management strategies, and access to technology with a patient-centered focus. Furthermore, technology platforms that streamline administrative burdens, tasks, and workflows, while offering actionable insights, can enable CRS to provide more robust patient care and manage trials more efficiently. Despite DCTs being promoted as a patient-centered approach to trials, our research suggests that significant progress is still needed to realize this vision. The focus on patient-facing technologies designed solely for data collection, which is understandably vital for clinical trials, and the shift toward remote, less frequent, and more limited CRS-patient interactions introduce new challenges for the overall patient trial experience. Relying on CRS to support the patient experience without adequately understanding each patient’s unique needs undermines their ability to fulfil this role. While DCTs and the technology solutions used show promise, they often fail to support key aspects of a patient-centered trial experience, potentially even detracting from it. A patient-centered experience extends beyond convenience and reduced travel, encompassing a complex interplay of personal, emotional, health, environmental, cultural, design, and contextual factors. Refocusing DCTs and how technology enhances these trials is necessary to support patients’ diverse and personalized needs with clear evidence of benefits. Prioritizing conditions and technologies that enhance the patient experience in DCTs while maintaining rigorous data collection standards can create a more balanced and effective approach to DCTs, ultimately benefiting both patients and CRS. This focus on both patient experience and trial staff support would further enhance the success of DCTs. Theoretically, DCTs are not merely a logistical innovation but a transformation of the traditional paradigms of clinical research. They necessitate a rethinking of the roles of technology, trial staff, and patients in achieving the conditions for scientific and experiential success. Future research should enable the integration of insights from sociotechnical systems theory, human-centered design, and patient-centered care, which can help refine the theoretical frameworks underpinning DCTs, ultimately shaping their future evolution.

## Appendix

Multimedia Appendix 1 Interview guide.

Multimedia Appendix 2 Reflexive thematic analysis process and outputs.

## Abbreviations

CRS: clinical research staff
DCT: decentralized clinical trial

## Data Availability

The datasets generated or analyzed during this study are not publicly available due to participants not granting consent to make the dataset available. In line with university guidelines and the participants’ signed consent, we have ensured that all data handling was conducted in accordance with ethical guidelines and confidentiality. We have provided some data excerpts in the study.
