# Supplementary material for: Adaptation of Clinical Research Staff to Decentralized Clinical Trials and Impacts on the Patient-Centered Experience: Qualitative Interview Study
Source: J Med Internet Res. 2025 Jun 16;27:e62947. doi: 10.2196/62947 (PMC12209732; doi:10.2196/62947)
Supplement: Multimedia Appendix 1 [file jmir_v27i1e62947_app1.docx]

**Topic 1 – Patient centred -**

**I would like to understand your thoughts the patient centred experience during trials**

**Q -** Think to your experience during a technology mediated setting (participants not on site as much, communication via tele health, data collection happening using apps) - What are the kinds of things that are done to ensure a patient centred and good experience and positive conditions for patients when the trial is Remote / DCT / technologically mediated?

*Follow up if needed*

- Q - Compare that to traditional site-based trials - how do you find that – is it the same or different
- Q – If you were to compare that to traditional trials – what do you think the differences are for site providing a patient centred experience
- Q – where there any benefits or challenges due to technology and the patient being remote on a trial
- Q - Is there specifically anything in the technology you or the patient uses create conditions to provide or help sites provide a patient centred experience / manage the patient centred experience – any tools or technologies or capabilities
- Q – what do you perceive the impacts of being on a remote trial are on the patient

**Q -** Think to your experience during a technology mediated setting (participants not on site as much, communication via tele health, data collection happening using apps) - How about from an Emotional and psychological well-being perspective during trials. What kind of things are done to support that perspective during Remote / DCT / technology mediated trials?

*Follow up if needed*

- Q - Compare that to traditional site-based trials - is it the same or different
- Q – where there any challenges in supporting the patient emotionally and psychologically due to technology and the patient being remote
- Q – what do you perceive the impacts of that are – on the patient
- Q - How do you know when a patient needs this support

**Topic 2 – Relationship**

**I would like to understand a little about relationship building with patients during technology trials**

**Q -** Think to your experience during a technology mediated setting (participants not on site as much, communication via tele health, patient data collection happening using apps). How are relationships built and maintained with patients when the trial is DCT / technologically mediated

*Follow up if needed*

- Q – If you were to compare that to traditional trials – what do you think the differences are for you and the patient building relationships – is it the same or different
- Q- So if you think of what you mentioned above regards the relationship what do you see the impacts of that are
- Q – How do you find those experiences in terms of building / maintaining relationships
- Q – How does the technology used in trials support this – the relationships between patient and site

**Topic 3 – Engagement**

**I would like to understand a little about engaging patients during technology trials**

**Q –** Think to your experience during a technology mediated trial (participants not on site as much, communication via tele health, patient data collection happening using apps). How are patients engaged or kept engaged with the trial during DCT / technology mediated trials?

*Follow up if needed*

- Q – what are some of the things you need to do at site with them that would encourage them to stay engaged
- Q – are there things the site does personally with individual patients (guidance -send them, talk to them about, remind them)
- Q – how do you know when a patient is not as engaged during the remote trial process
- Q – compare that to traditional trials what’s the same and what’s different
- Q – Is there specifically anything in the technology you or the patient uses create conditions to provide or help sites provide a patient / manage to keep patients engaged – any tools or technologies or capabilities

**Final - Ending question**

**Q –** Based on your experiences what do you feel have been the overall effects of technology and the remote nature on the patient receiving a truly patient centred experience ?
