# Supplementary material for: Adaptation of Clinical Research Staff to Decentralized Clinical Trials and Impacts on the Patient-Centered Experience: Qualitative Interview Study
Source: J Med Internet Res. 2025 Jun 16;27:e62947. doi: 10.2196/62947 (PMC12209732; doi:10.2196/62947)
Supplement: Multimedia Appendix 2 [file jmir_v27i1e62947_app2.docx]

**Supplementary Material 2**

Reflexive Thematic Data Analysis Process and Outputs

This supplementary material provides visual representation and examples of the phases of the data analysis process following the Braun and Clarke Reflexive Thematic Analysis (RTA) methodology. The content spans across key phases of data analysis, showcasing various progression including initial coding, refined coding, and theme development.

**Appendix: 1 – 9**

Screen grabs of examples of initial codes in Excel and refined codes NVivo, showcasing data broken down into themes and sub-themes, generated through the coding methodology and the process of reflection and refinement during phases 2 and 4 of data analysis in the Braun and Clarke reflexive thematic analysis (RTA) process.

**Appendix: 10 - 12**

Screen grabs and close up grabs from Miro of the research and examples of sorting, highlighting early conceptualisation in the development of themes and sub-themes during phases 3 and 4 of data analysis in the Braun and Clarke (RTA) process.

**Appendix: 13**

Photos from the research team workshop on theme finalisation, part of phase 4 in the Braun and Clarke Data Analysis (RTA) process.

**Appendix: 14**

Final set of themes and sub-themes developed as part of phase 5 of data analysis in the Braun and Clarke reflexive thematic analysis (RTA) process.

**Appendix 1 – 9**

Screen grabs of examples of initial codes in Excel and refined codes NVivo, showcasing data broken down into themes and sub-themes, generated through the coding methodology and the process of reflection and refinement during phases 2 and 4 of data analysis in the Braun and Clarke reflexive thematic analysis (RTA) process.

Appendix 1


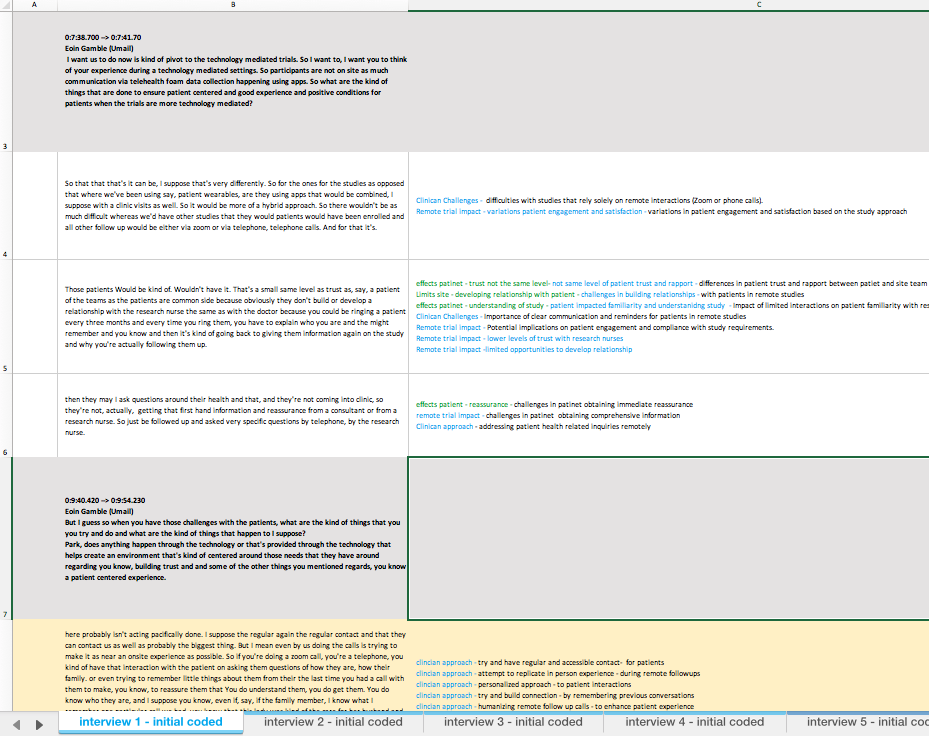


Appendix 2


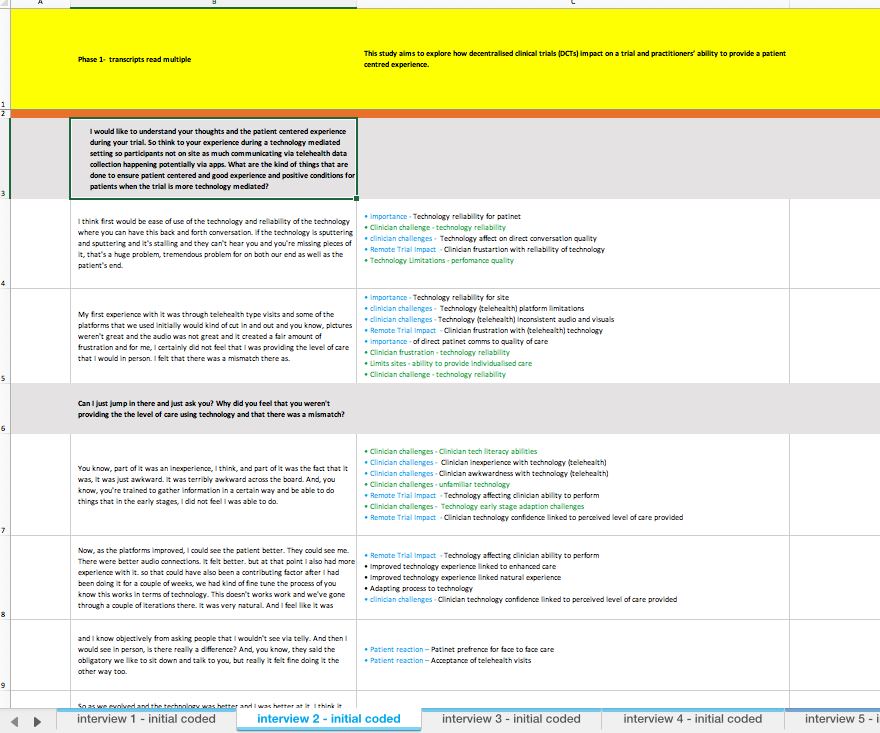


Appendix 3


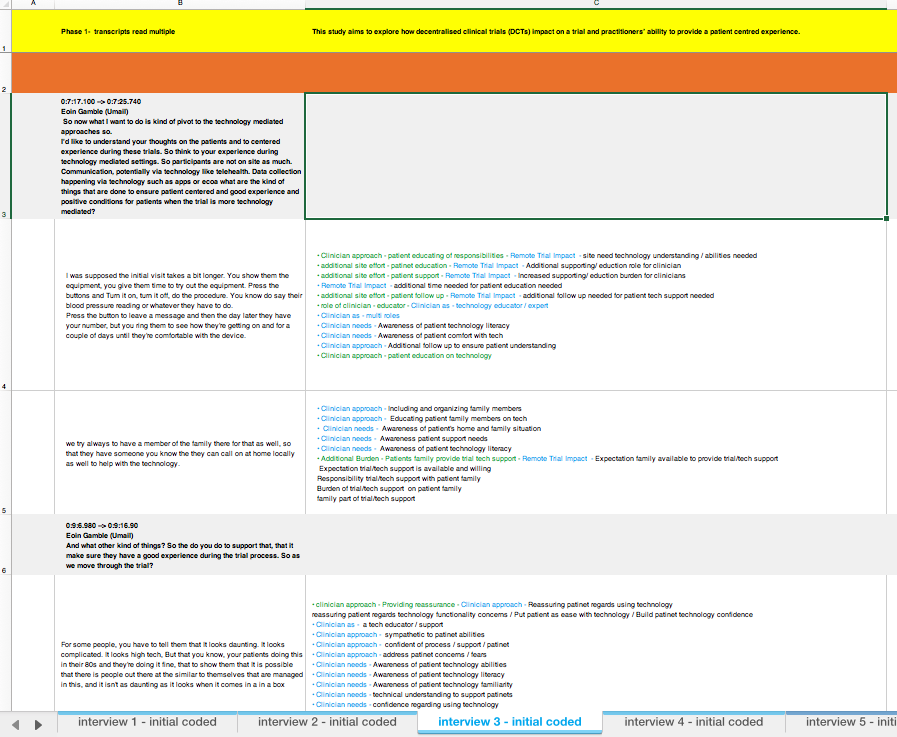


Appendix 4

Theme 1 screen grab of Nvivo coding and excerpts


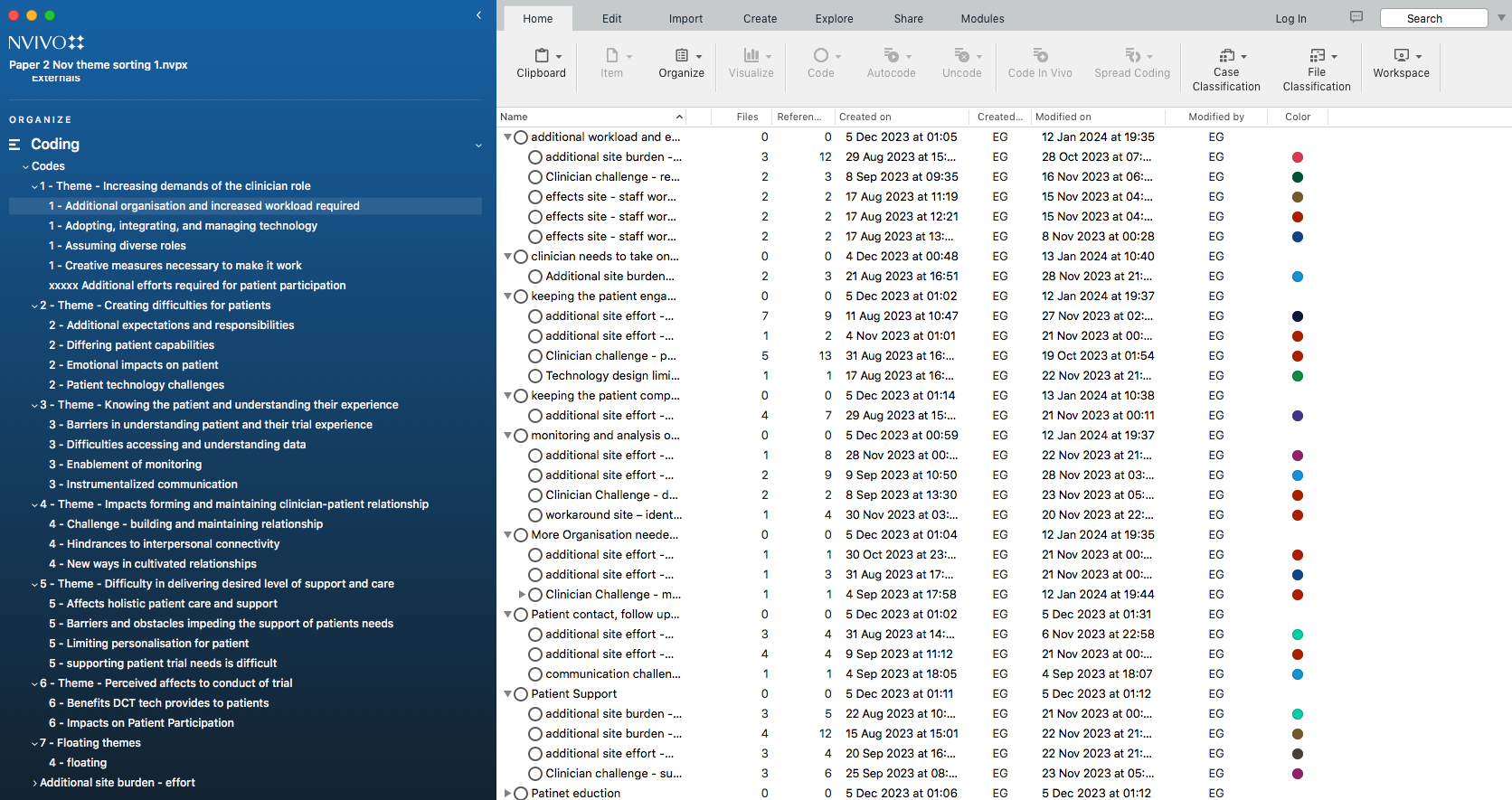


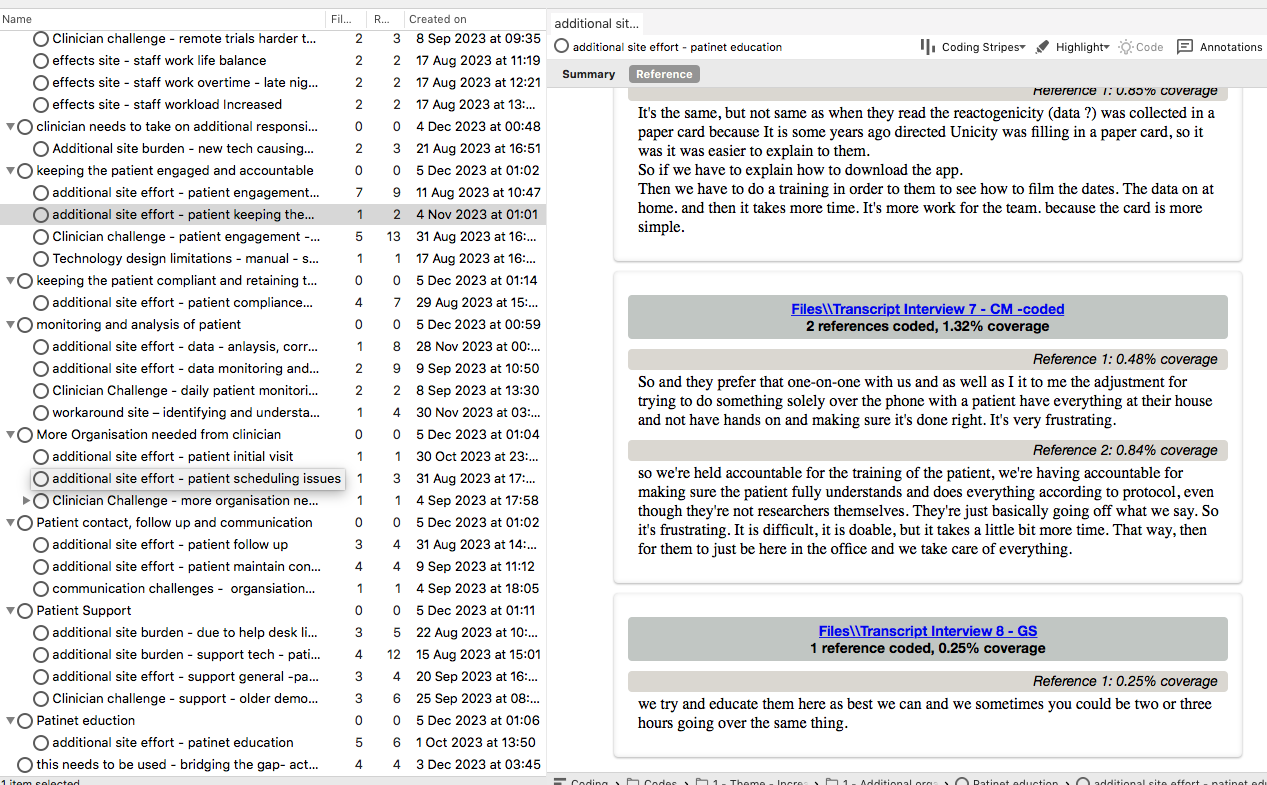


Appendix 5

Theme 2 screen grab of Nvivo coding and excerpts


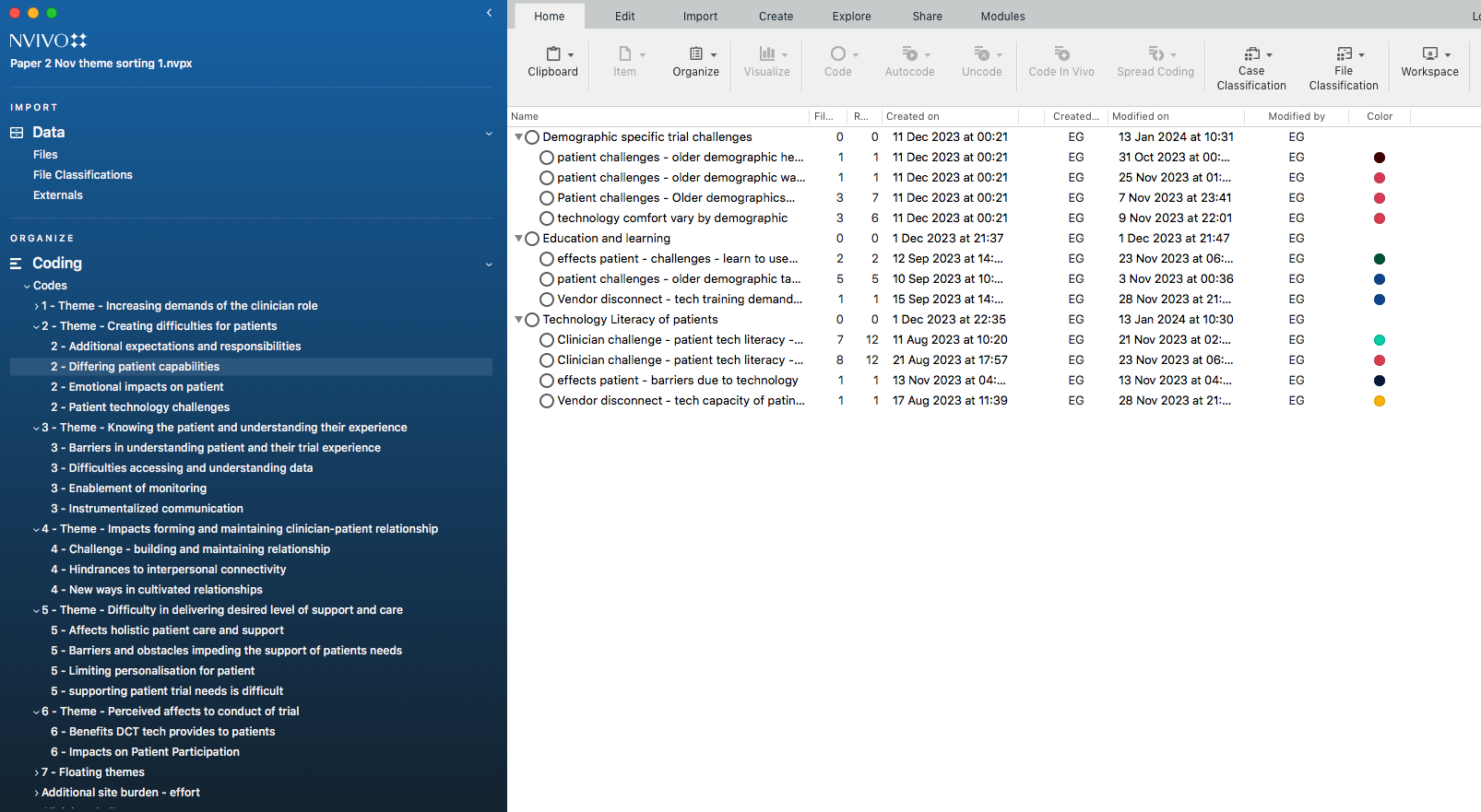


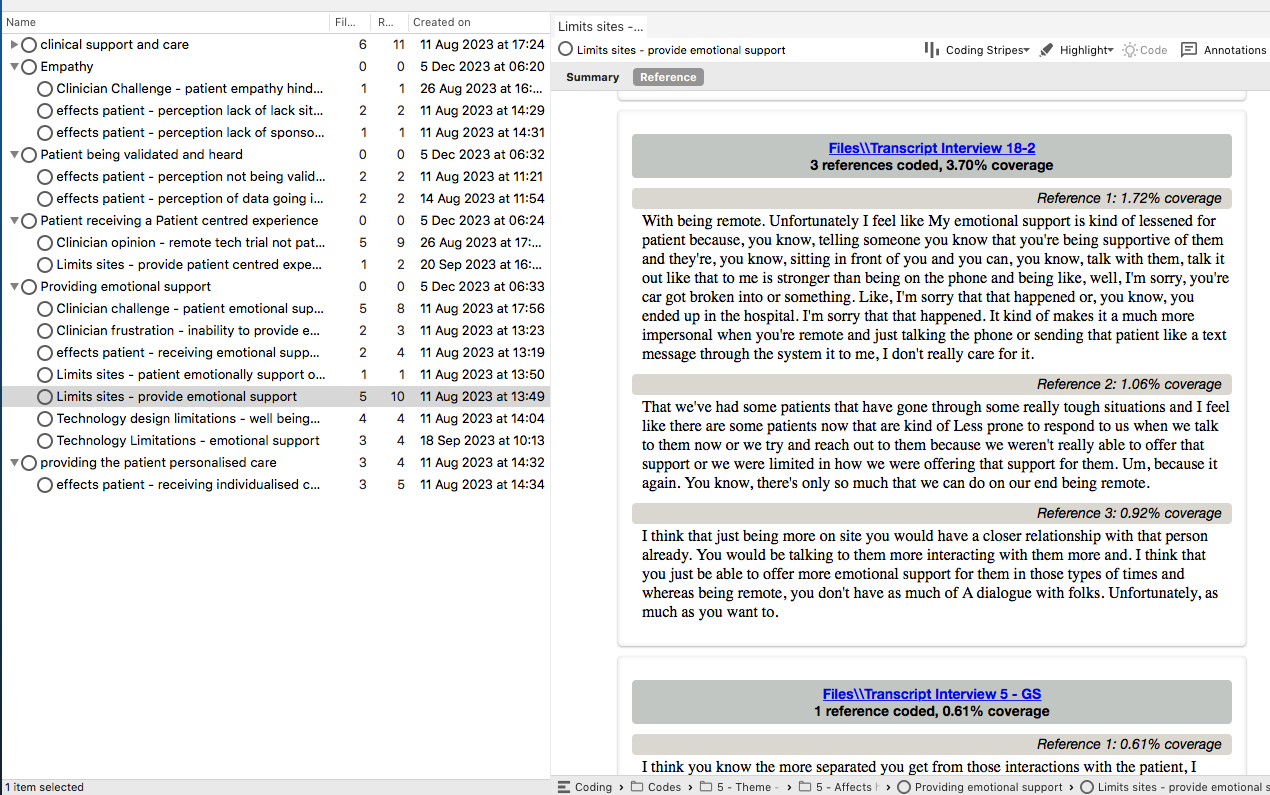


Appendix 6

Theme 3 screen grab of Nvivo coding and excerpts


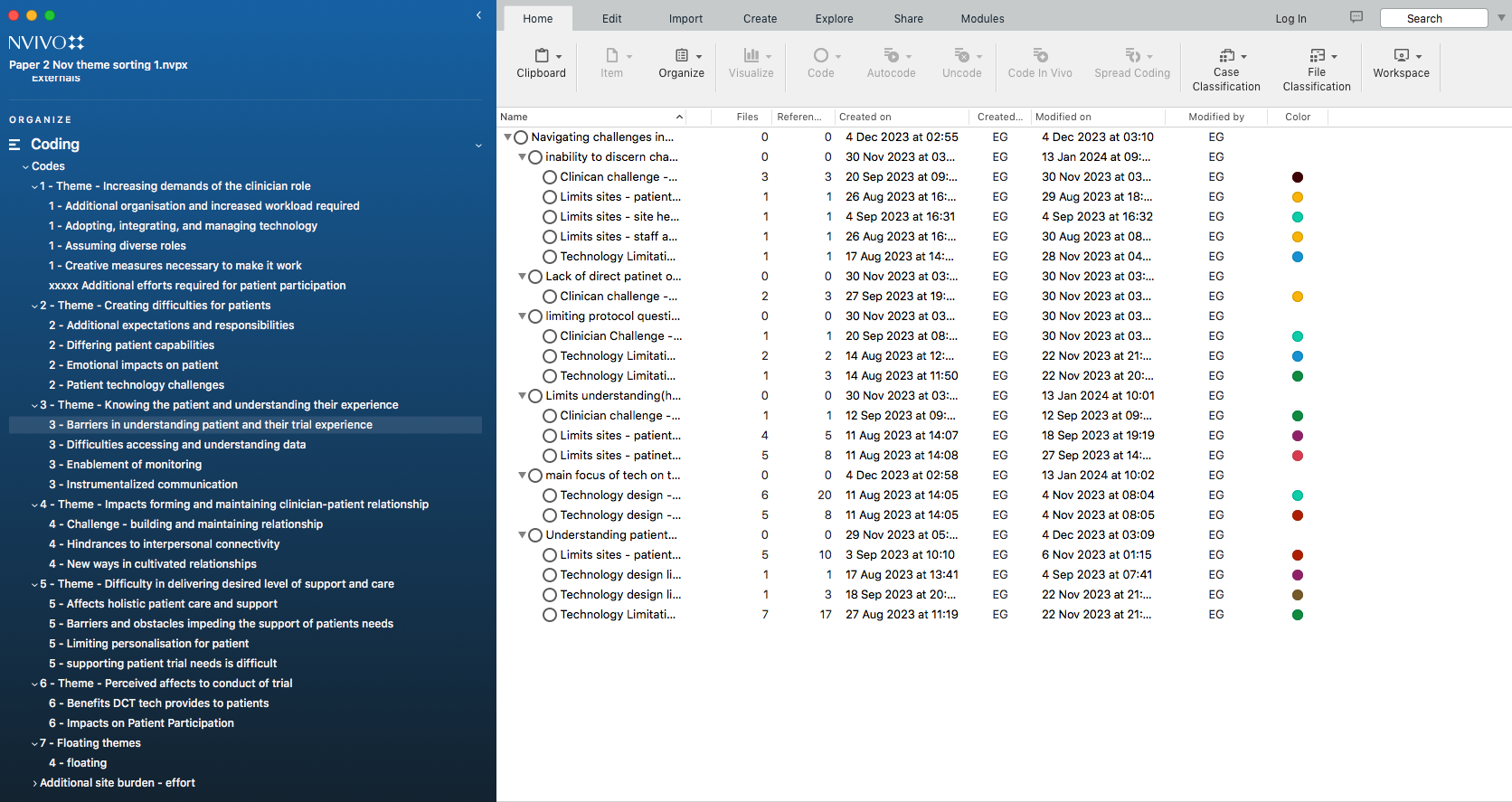


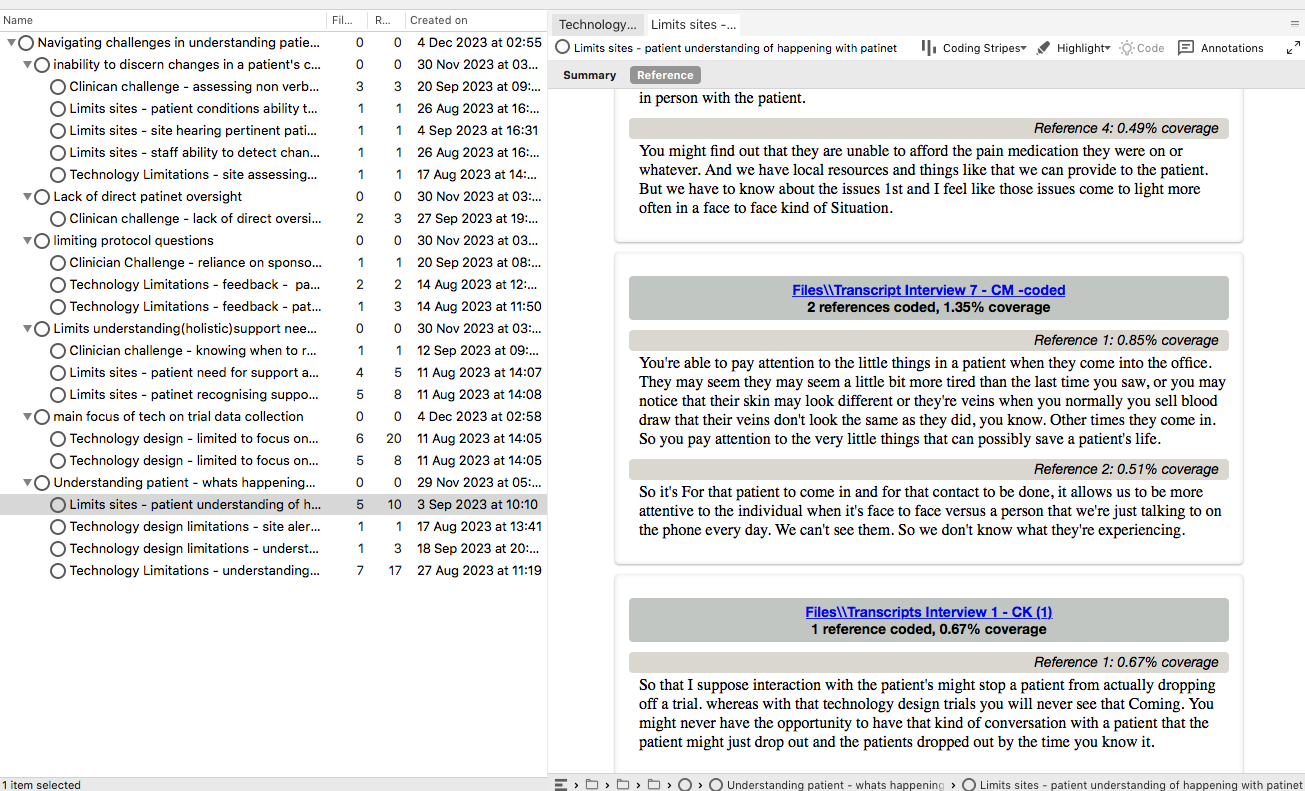


Appendix 7

Theme 4 screen grab of Nvivo coding and excerpts


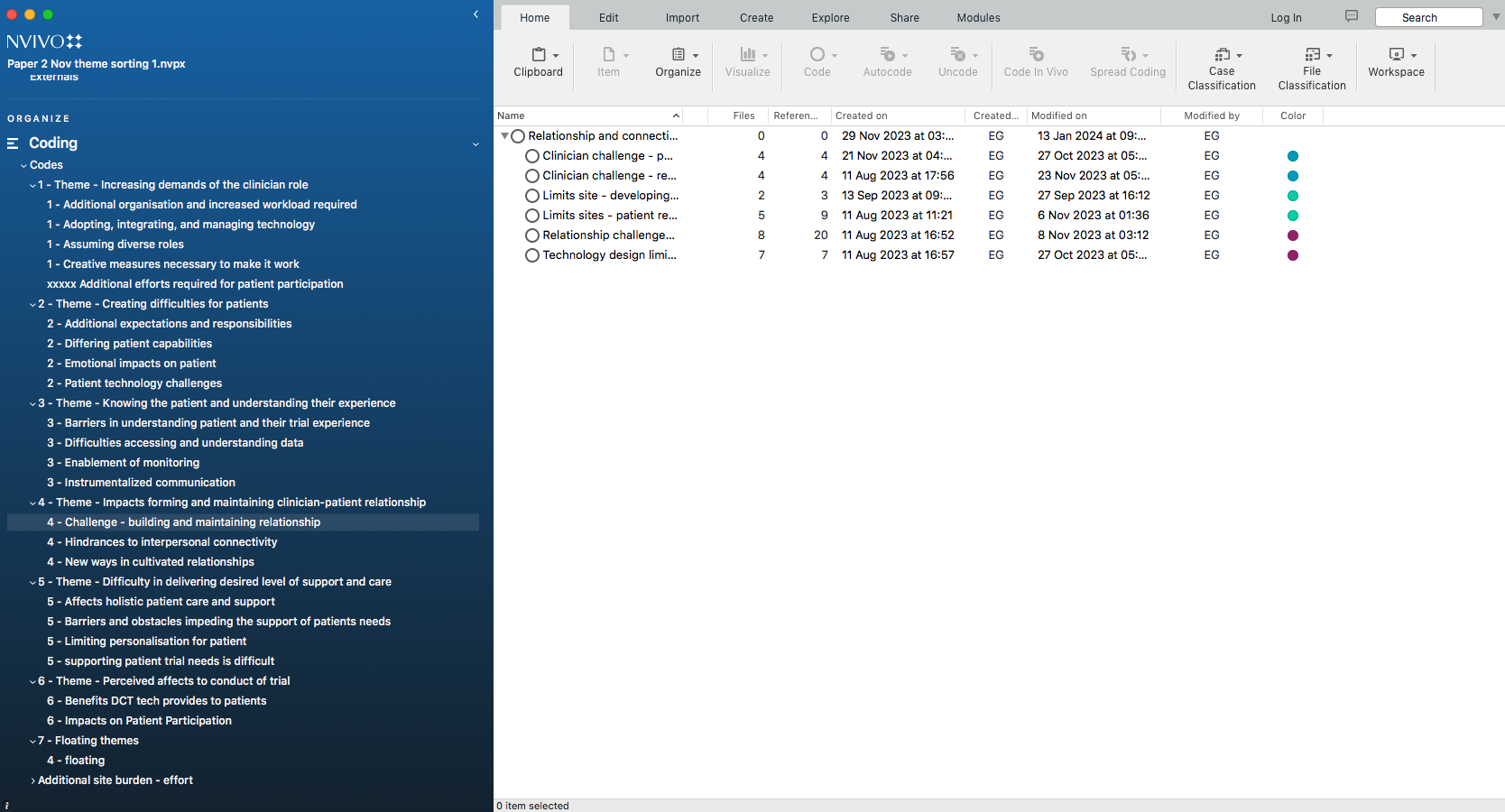


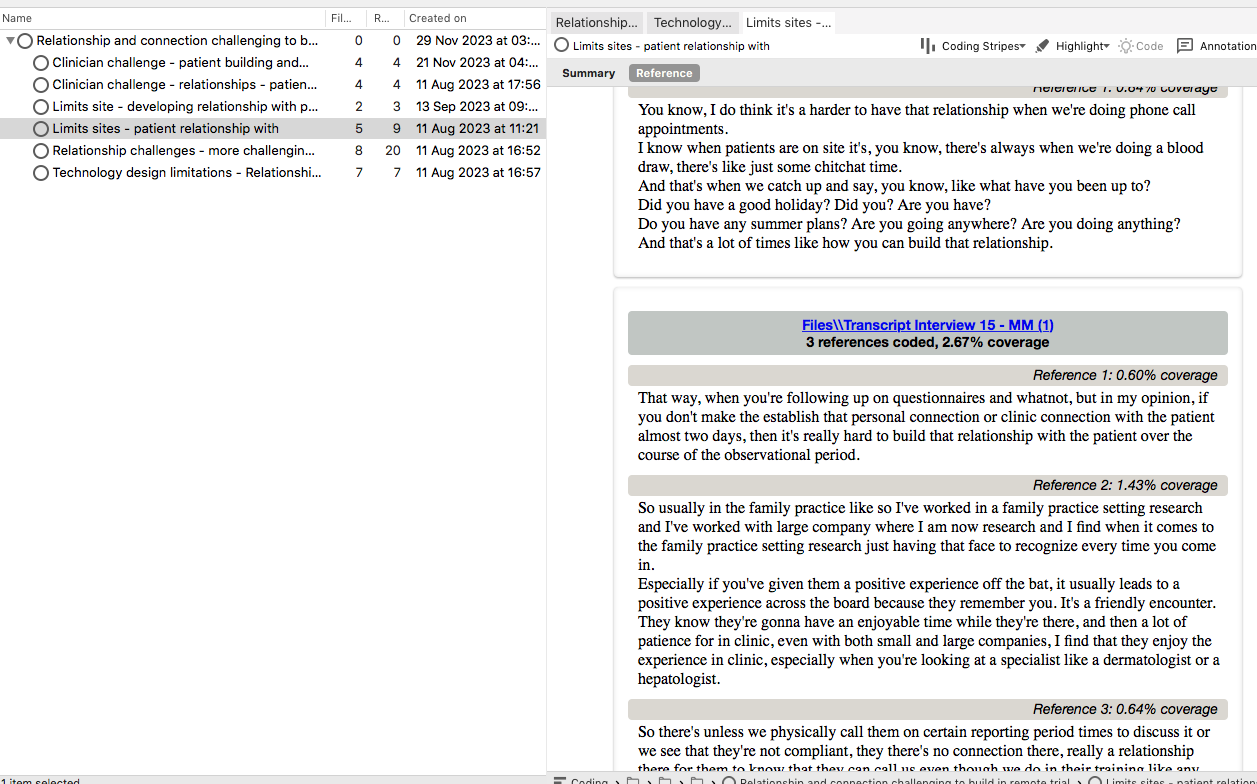


Appendix 8

Theme 5 screen grab of Nvivo coding and excerpts


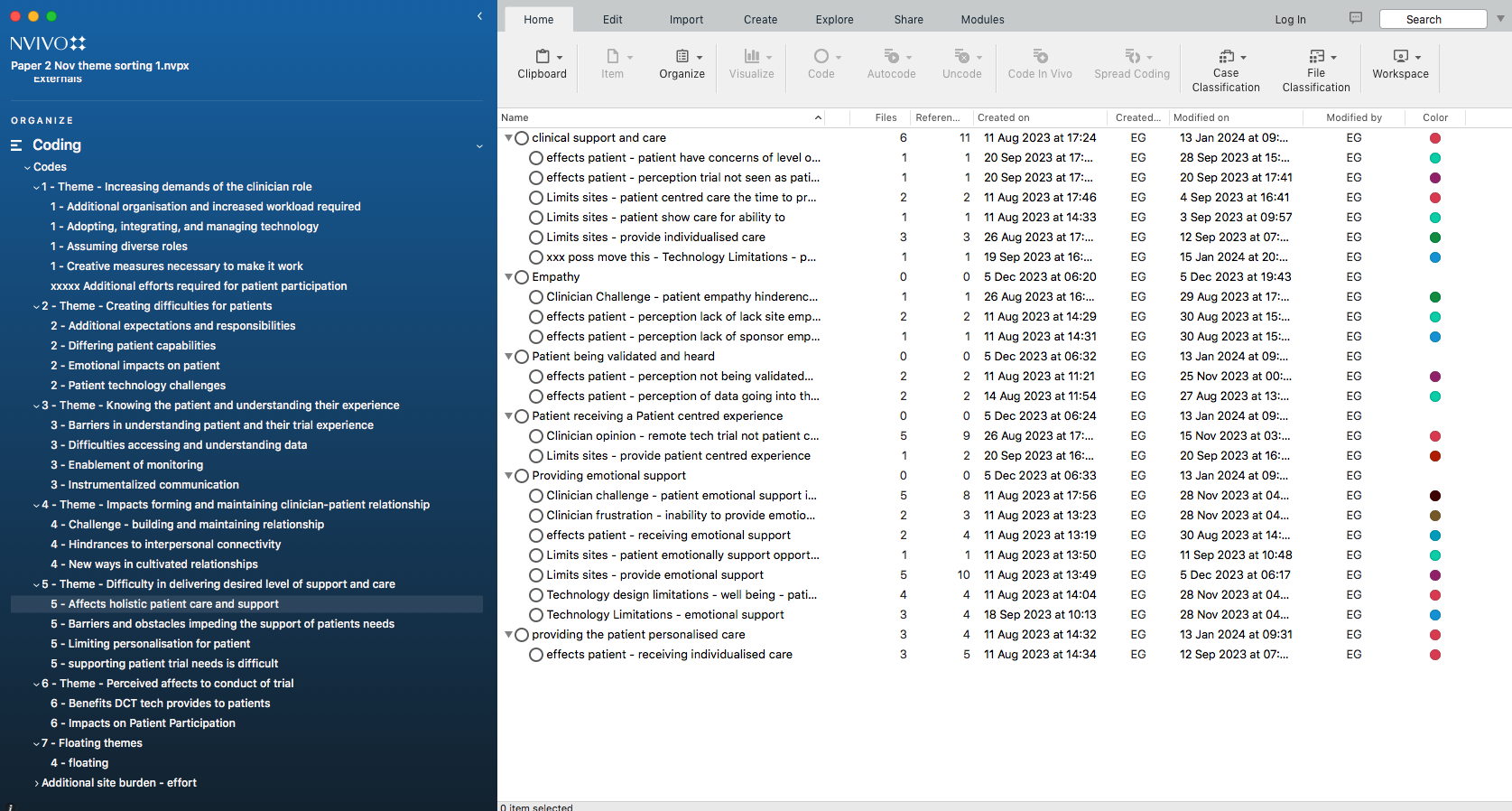


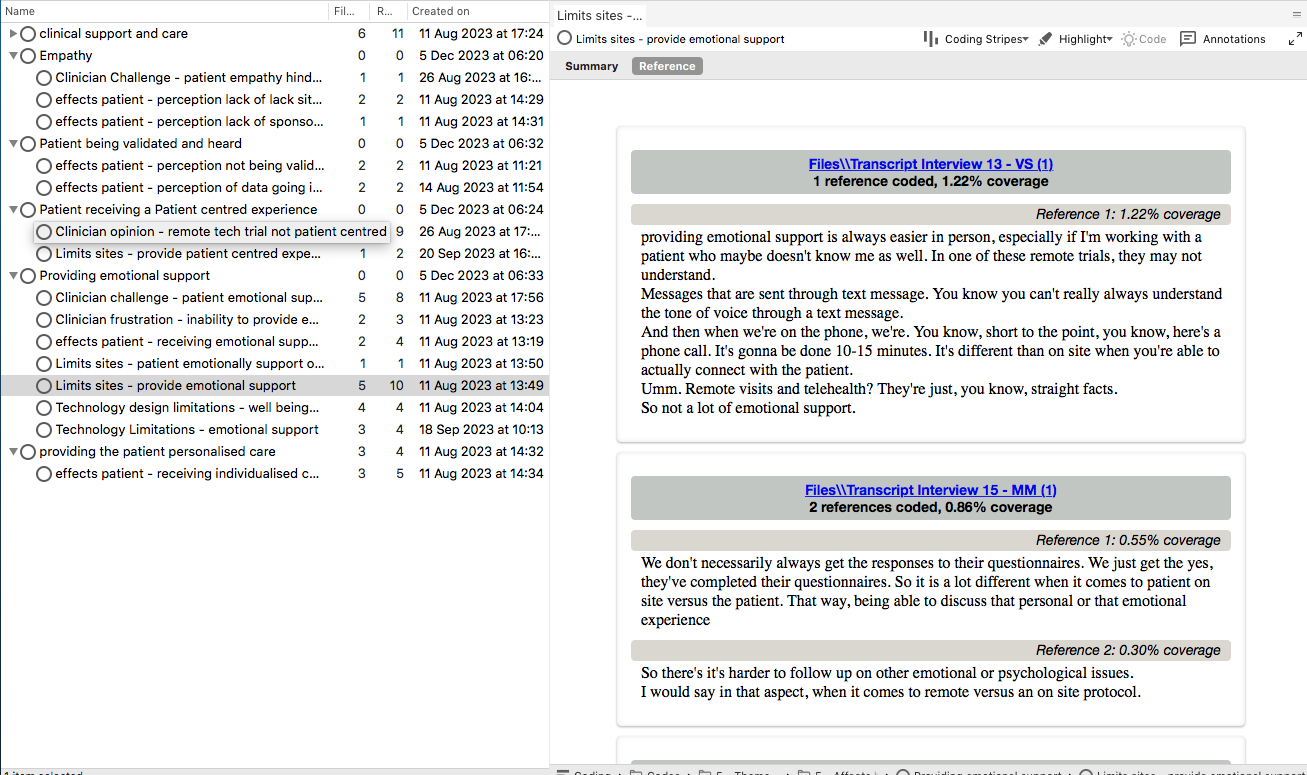


Appendix 9

Theme 6 screen grab of Nvivo coding and excerpts


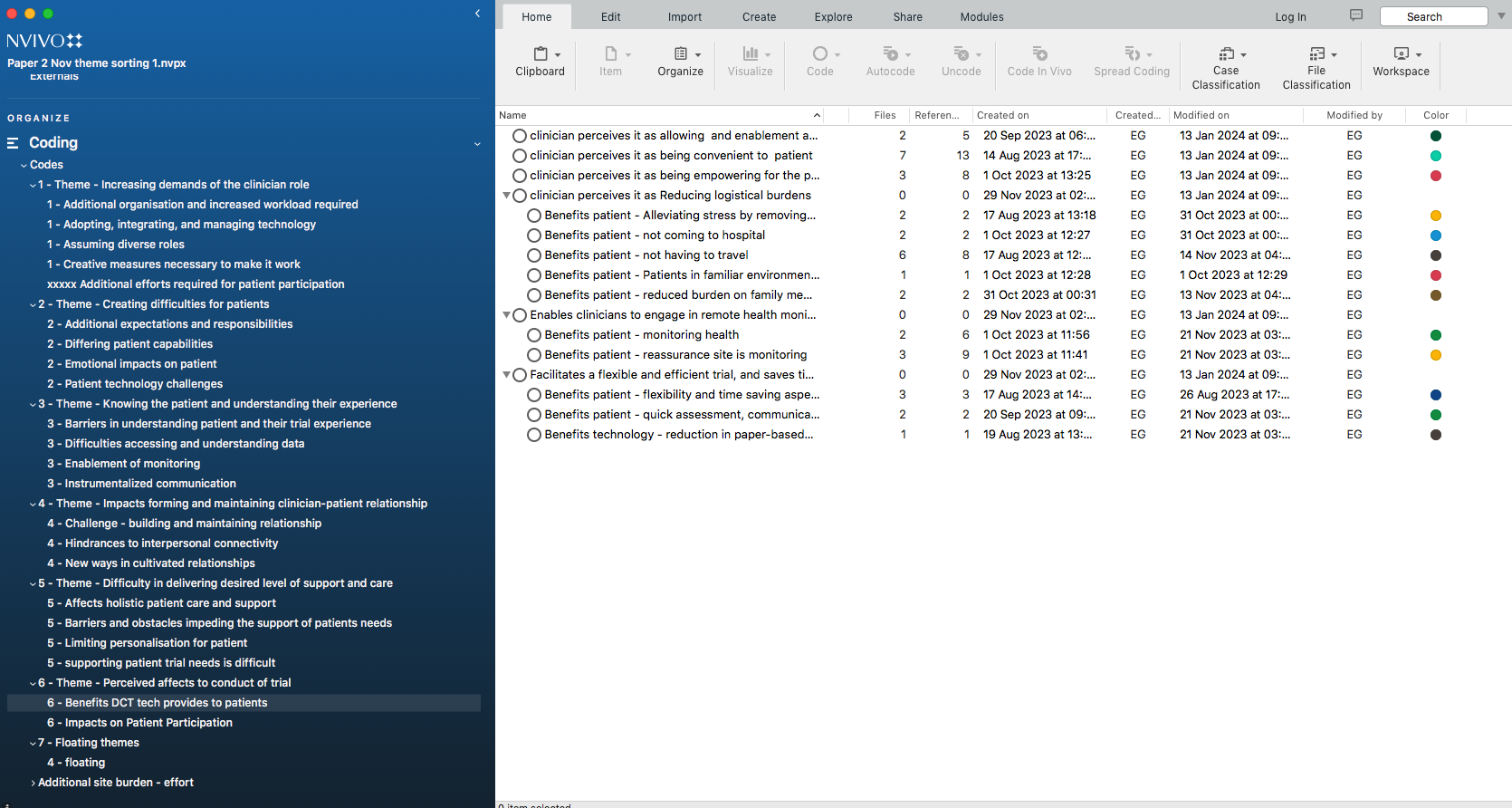


**
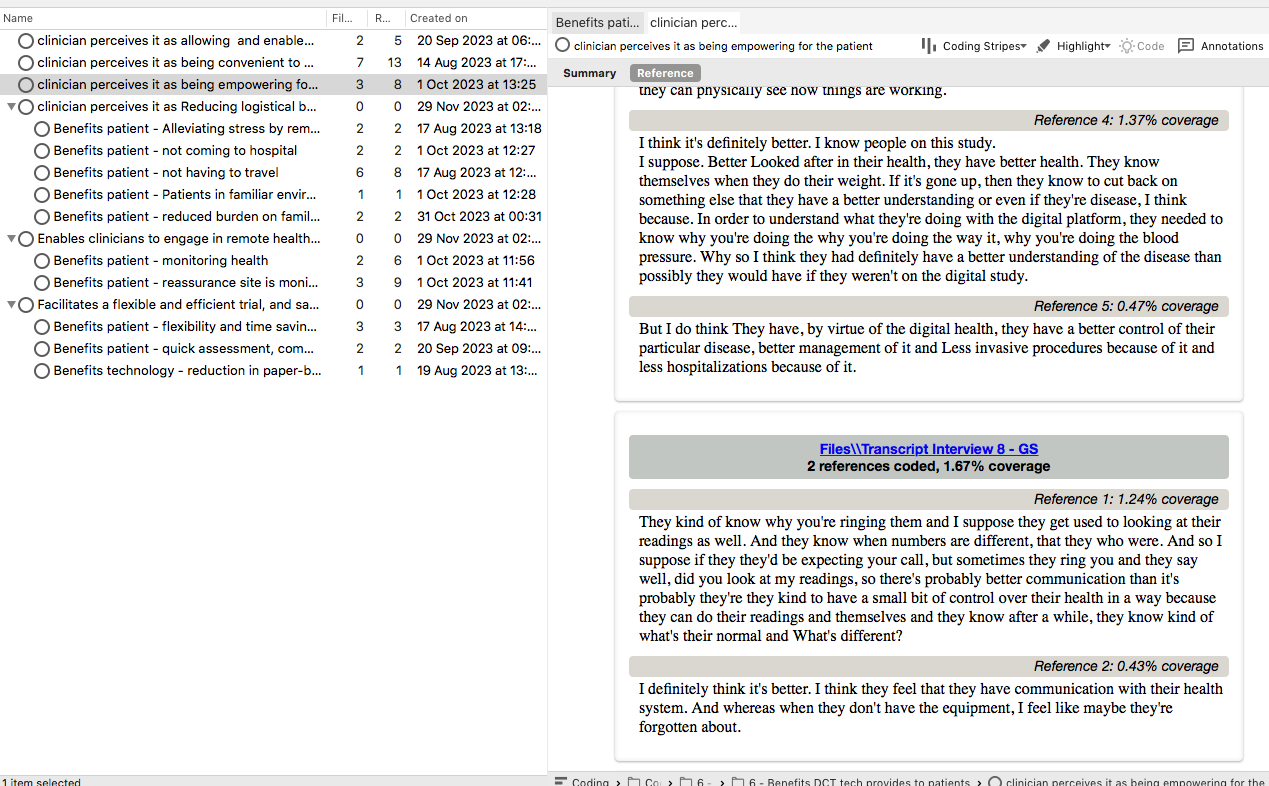
**

**Appendix 10 – 14**

Miro screen grabs from the research and examples at various stages of sorting, highlighting conceptualisation in the development of themes and sub-themes during phases 3 and 4 of data analysis in the Braun and Clarke reflexive thematic analysis (RTA) process.

Appendix 10


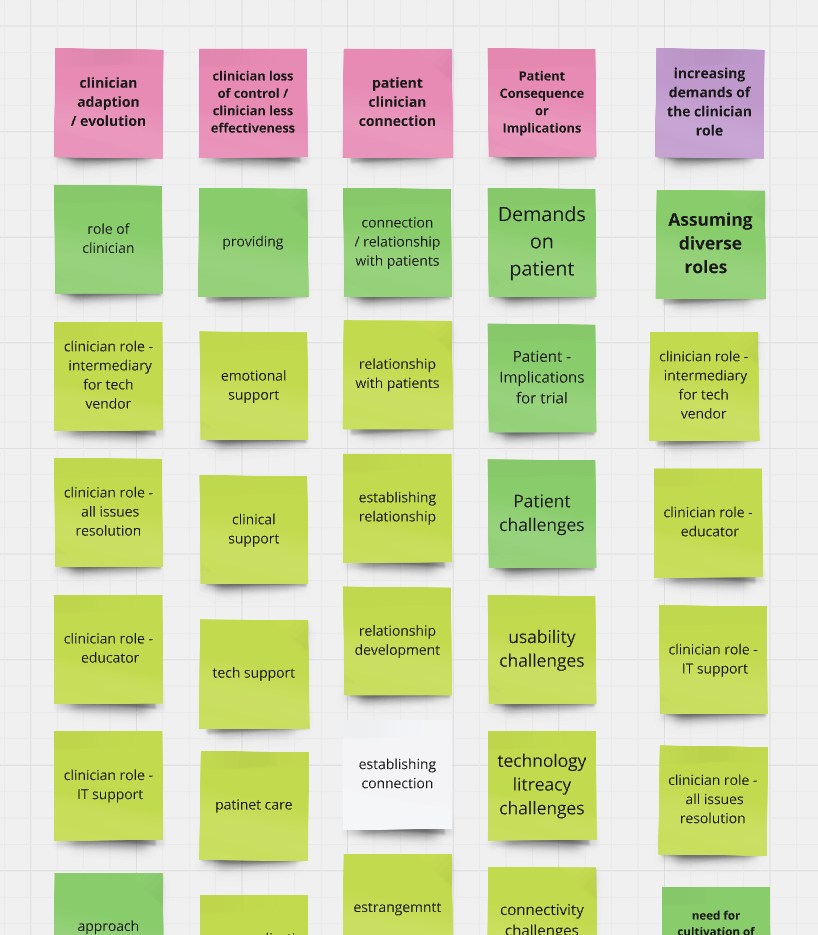


Detail grab of screen grab

Appendix 11


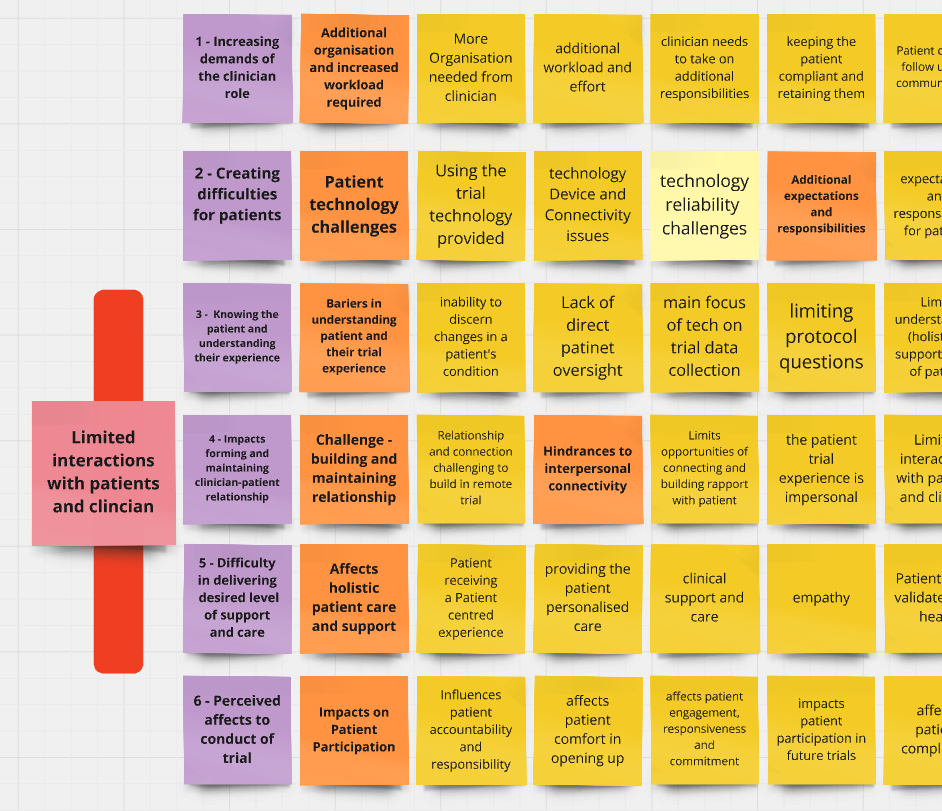


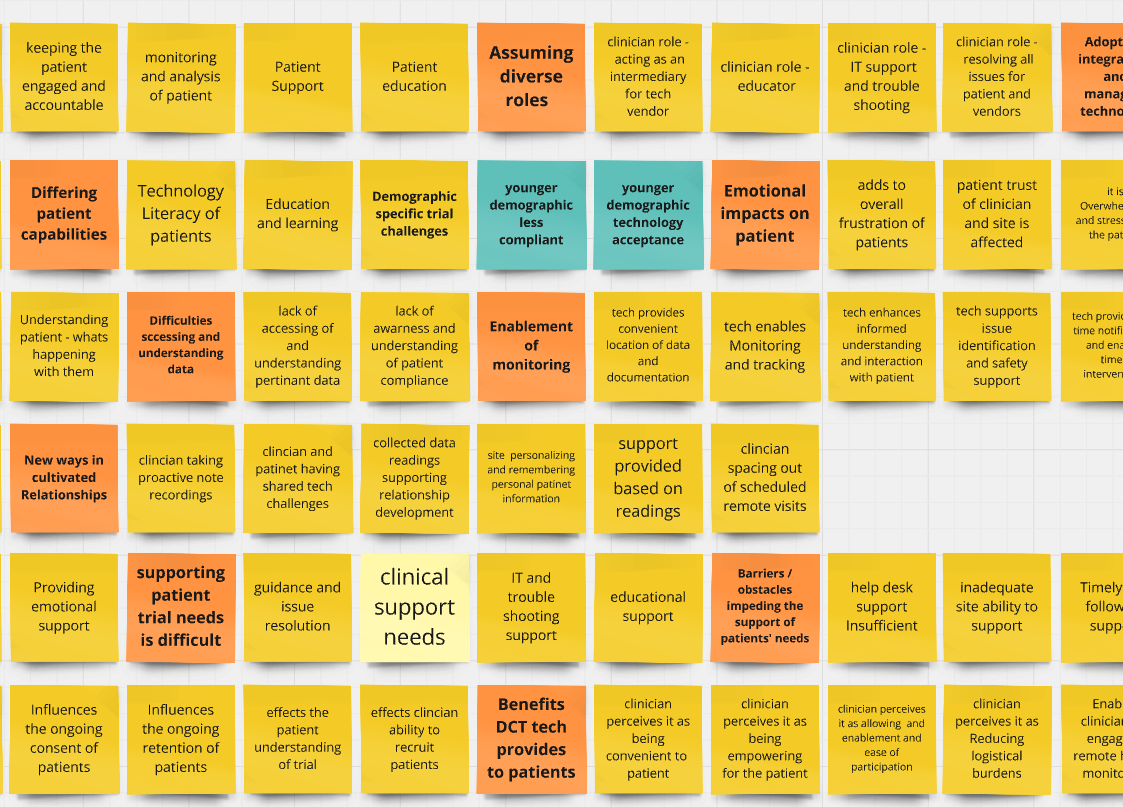


Detail of screen grab

Appendix 12


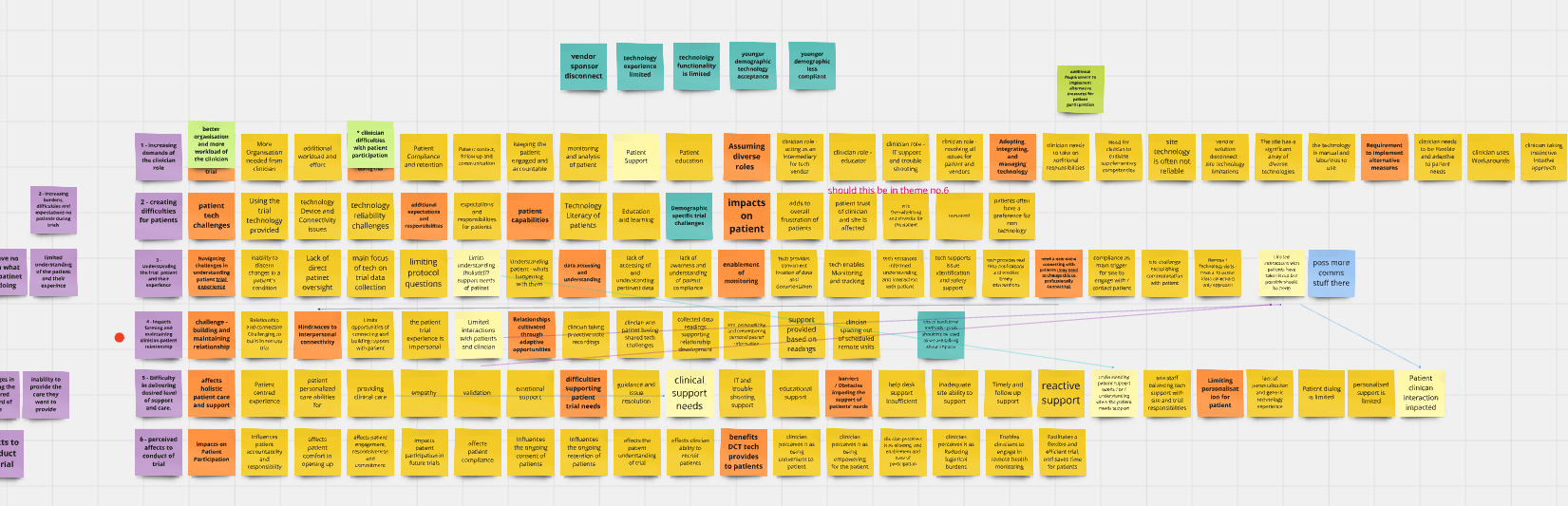


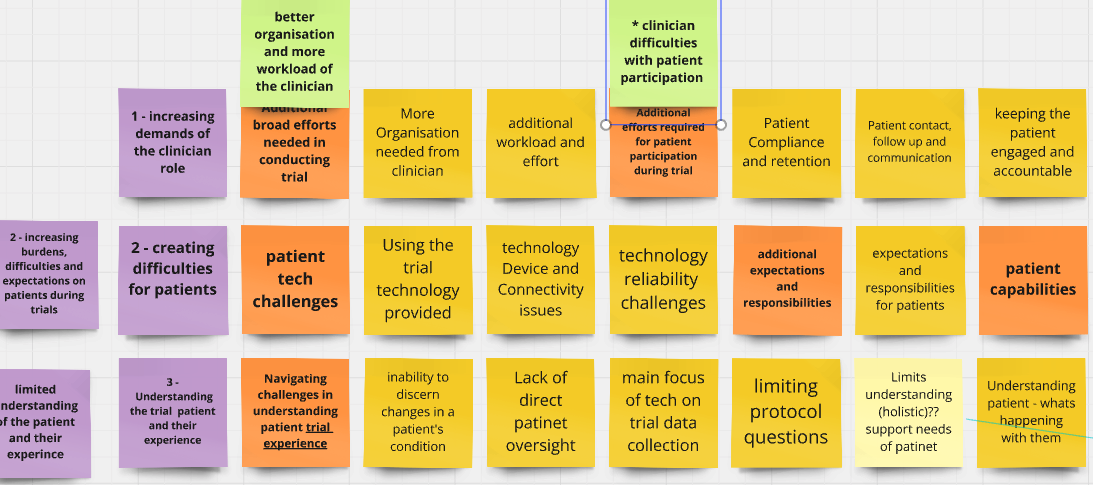


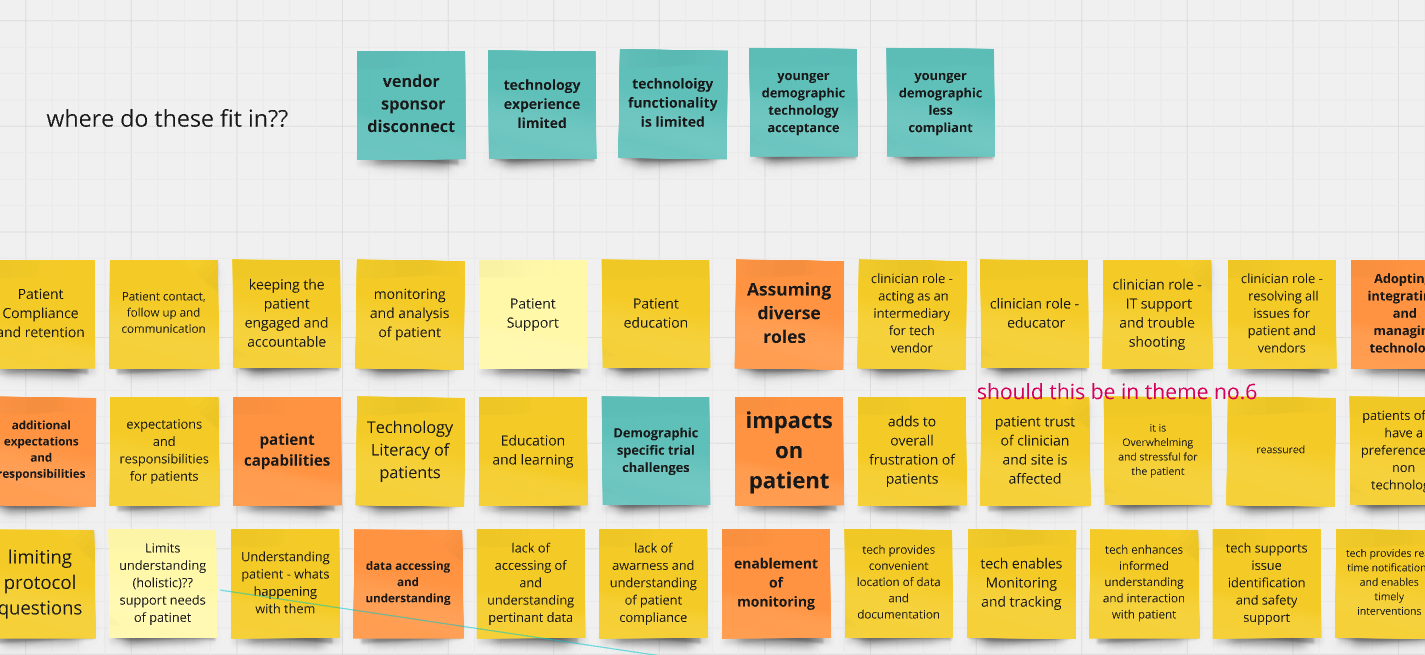


Detail of screen grab

**Appendix 13:**

Photos from the research team workshop on theme finalisation, part of phase 4 in the Braun and Clarke reflexive thematic analysis (RTA) process.


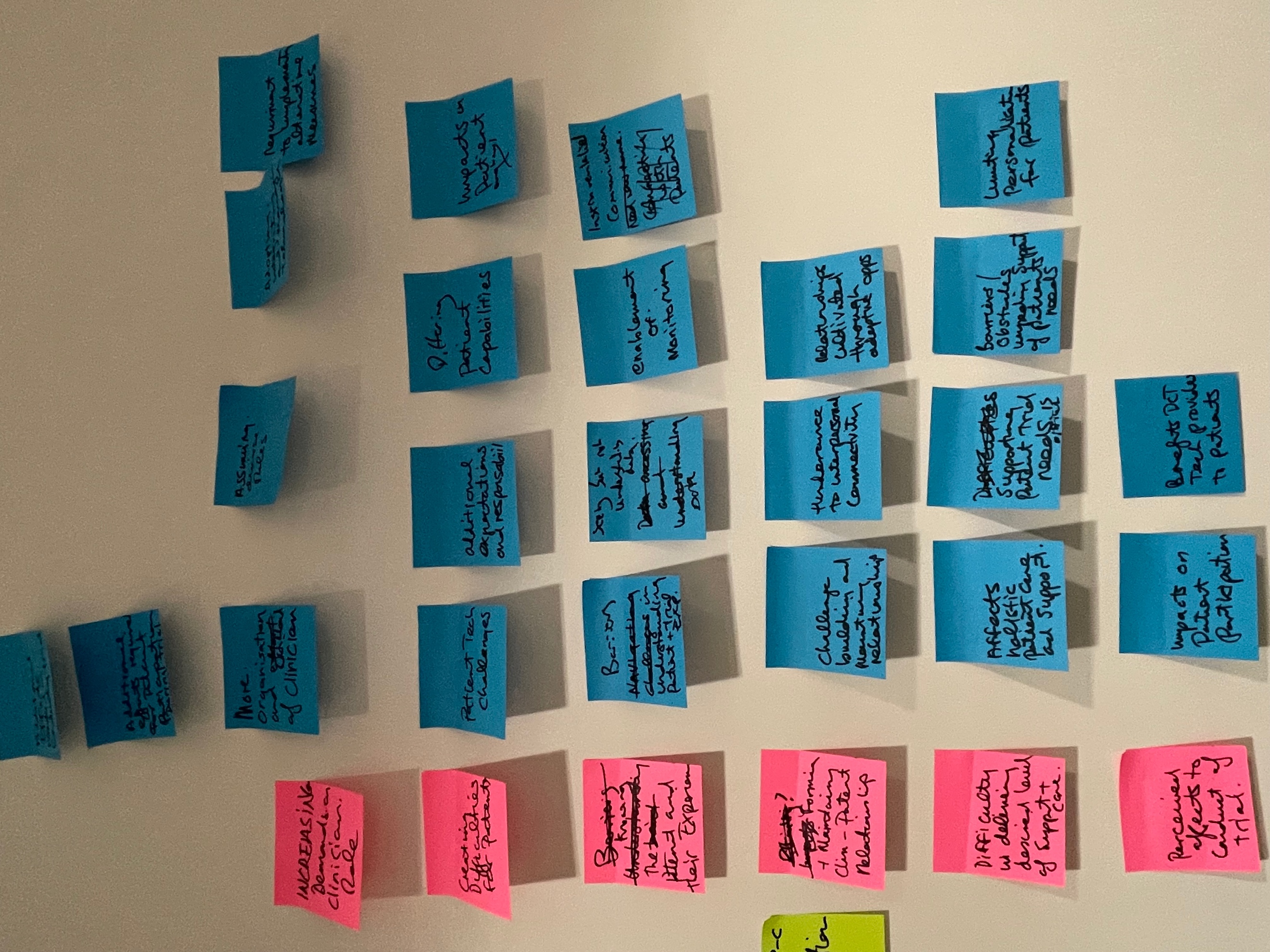

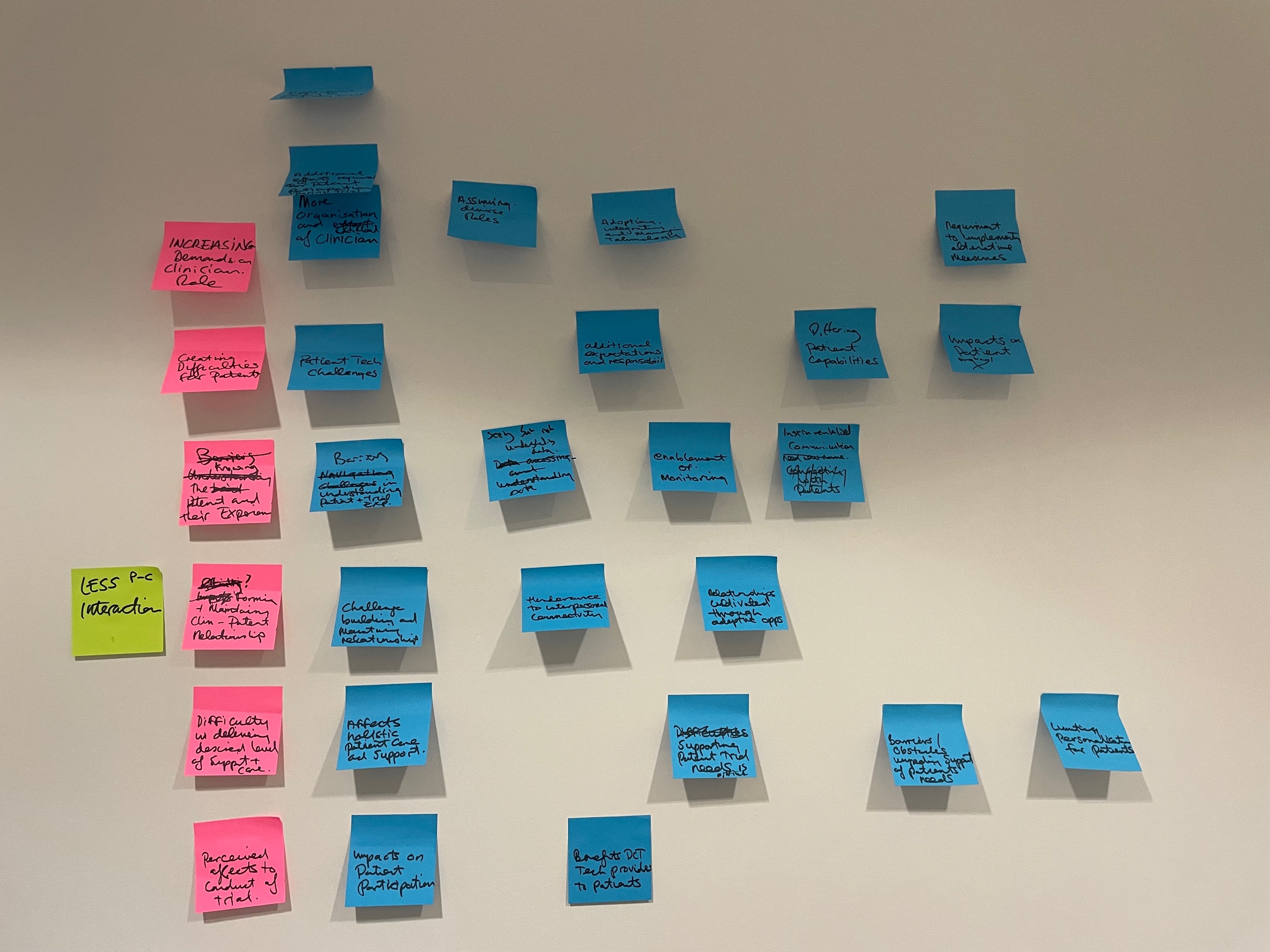


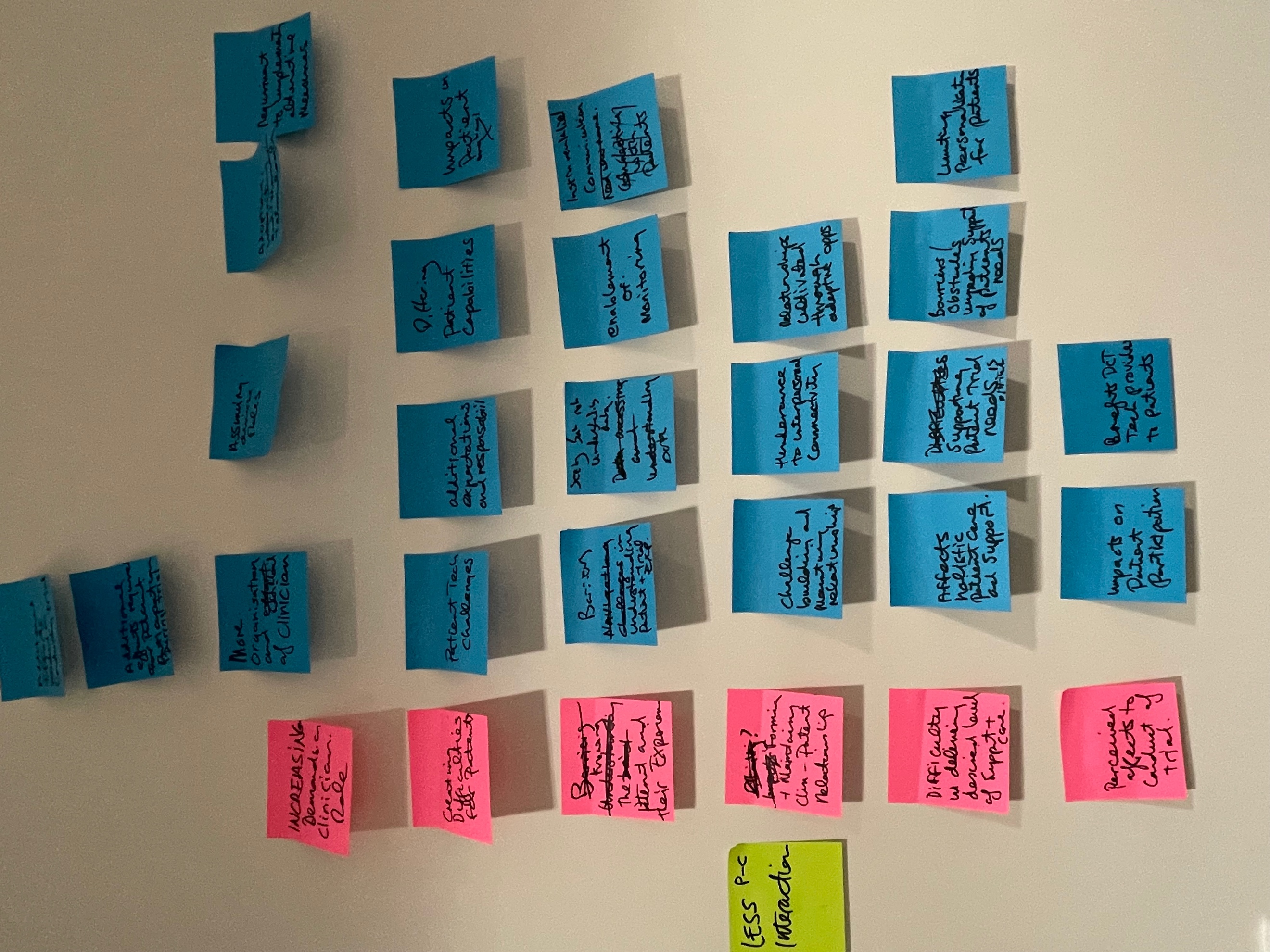


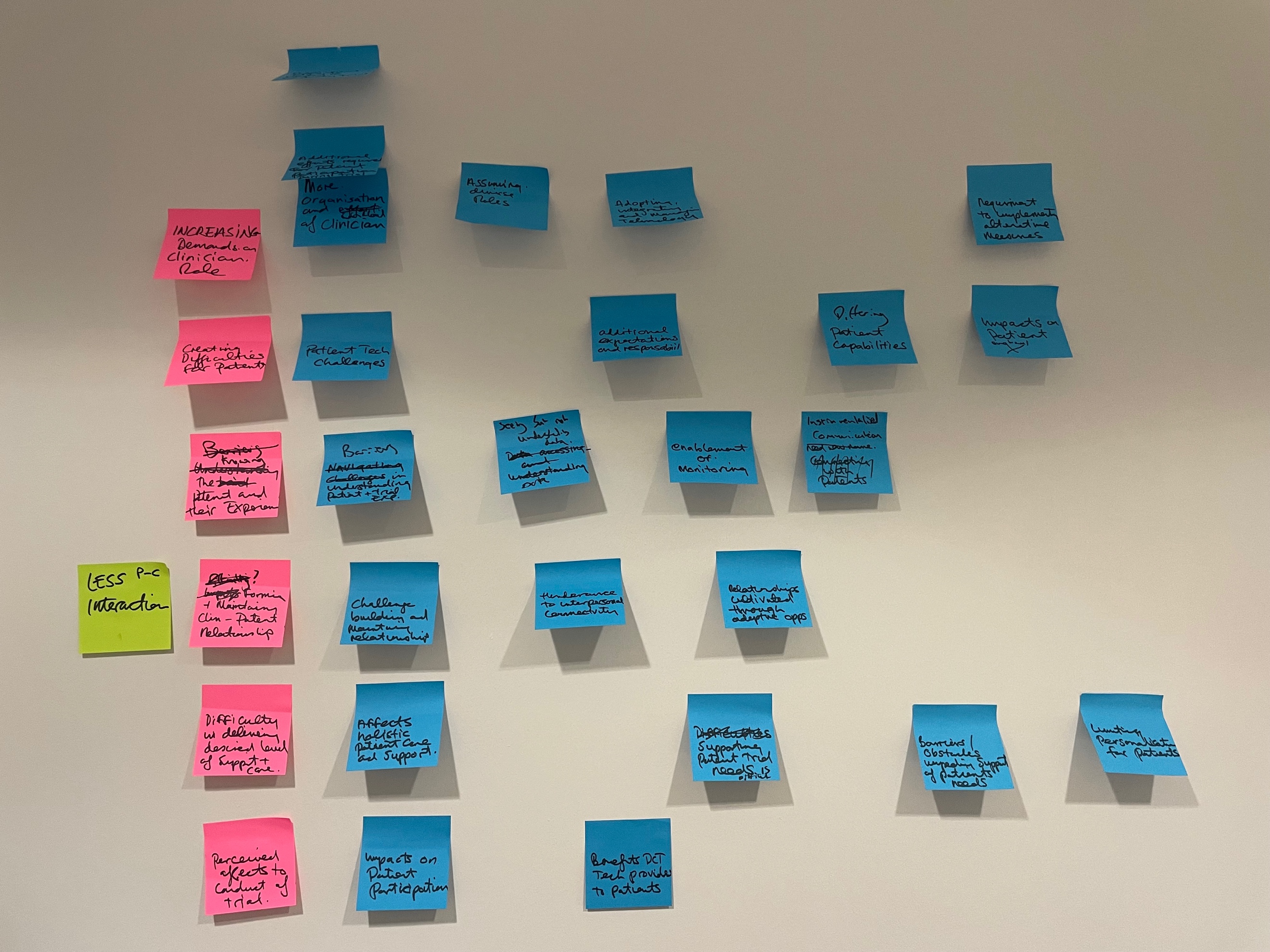


**Appendix 14:**

Final set of themes and sub-themes developed as part of phase 5 of data analysis in the Braun and Clarke reflexive thematic analysis (RTA) process.

**
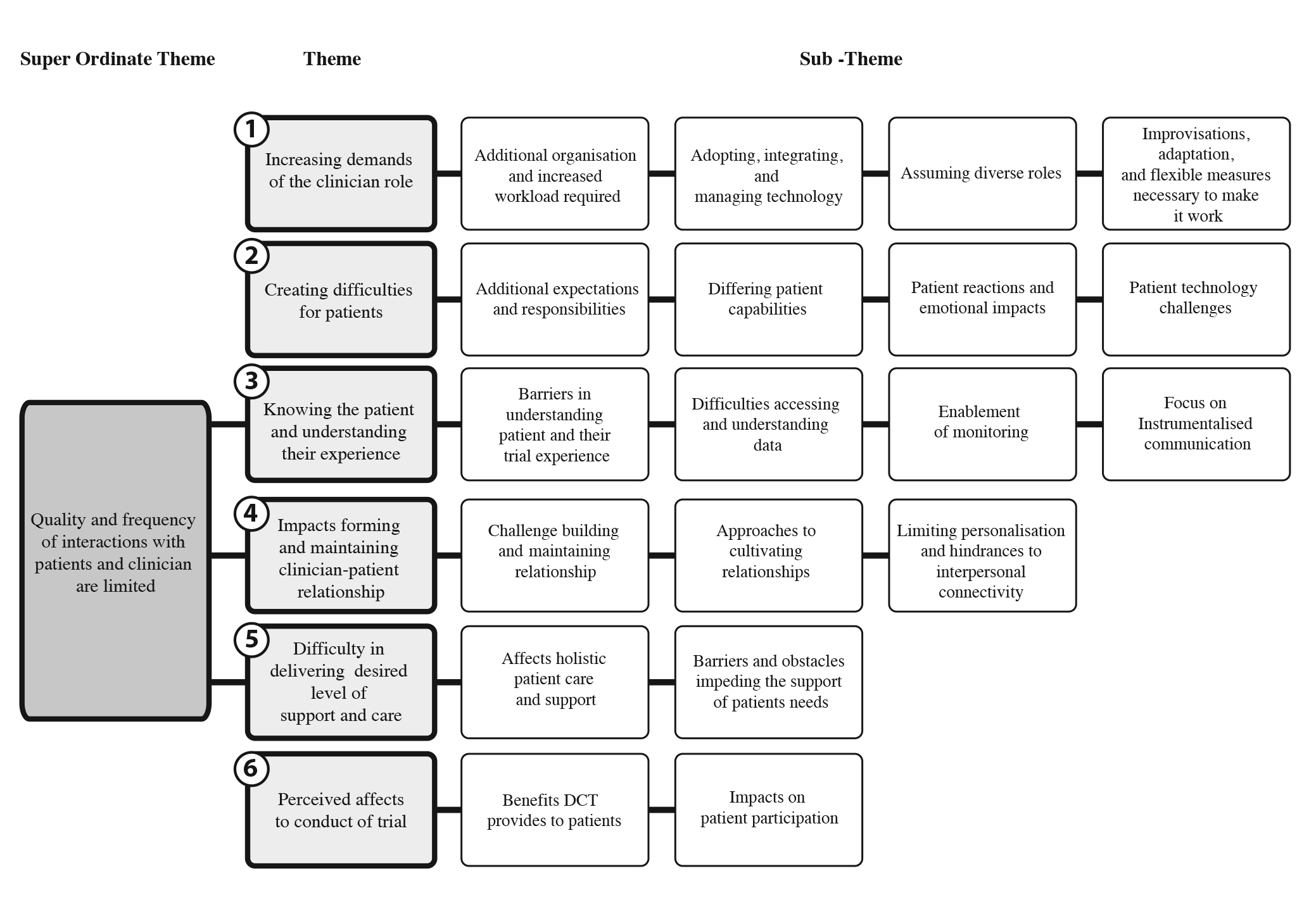
**
